# Genetic regulation of human brain proteome reveals proteins implicated in psychiatric disorders

**DOI:** 10.1038/s41380-024-02576-8

**Published:** 2024-05-09

**Authors:** Jie Luo, Ling Li, Mingming Niu, Dehui Kong, Yi Jiang, Suresh Poudel, Annie W. Shieh, Lijun Cheng, Gina Giase, Kay Grennan, Kevin P. White, Chao Chen, Sidney H. Wang, Dalila Pinto, Yue Wang, Chunyu Liu, Junmin Peng, Xusheng Wang

**Affiliations:** 1 State Key Laboratory for Managing Biotic and Chemical Threats to the Quality and Safety of Agro‐products, Zhejiang Academy of Agricultural Sciences, Hangzhou, Zhejiang 310021 China; 2 Department of Genetics, Genomics & Informatics, University of Tennessee Health Science Center, Memphis, TN 38103 USA; 3 Department of Structural Biology and Department of Developmental Neurobiology, St. Jude Children’s Research Hospital, Memphis, TN 38105 USA; 4 Department of Epidemiology and Biostatistics, School of Public Health, Tongji Medical College, Huazhong University of Science and Technology, Wuhan, Hubei 430030 China; 5 Center for Proteomics and Metabolomics, St. Jude Children’s Research Hospital, Memphis, TN 38105 USA; 6 Knapp Center for Biomedical Discovery, University of Chicago, Chicago, IL 60637 USA; 7 Department of Biochemistry and Precision Medicine, National University, Singapore, 119077 Singapore; 8 Center for Medical Genetics and Human Key Laboratory of Medical Genetics, School of Life Sciences, Central South University, Changsha, Hunan 410083 China; 9 Center for Human Genetics, Brown Foundation Institute of Molecular Medicine, The University of Texas Health Science Center at Houston, Houston, TX 77225 USA; 10 Department of Genetics and Genomic Sciences, Icahn School of Medicine at Mount Sinai, New York, NY 10029 USA; 11 Department of Electrical and Computer Engineering, Virginia Polytechnic Institute and State University, Arlington, VA 22203 USA; 12 Department of Psychiatry, SUNY Upstate Medical University, Syracuse, NY 13210 USA

**Keywords:** Genetics, Neuroscience, Schizophrenia

## Abstract

Psychiatric disorders are highly heritable yet polygenic, potentially involving hundreds of risk genes. Genome-wide association studies have identified hundreds of genomic susceptibility loci with susceptibility to psychiatric disorders; however, the contribution of these loci to the underlying psychopathology and etiology remains elusive. Here we generated deep human brain proteomics data by quantifying 11,608 proteins across 268 subjects using 11-plex tandem mass tag coupled with two-dimensional liquid chromatography-tandem mass spectrometry. Our analysis revealed 788 *cis*-acting protein quantitative trait loci associated with the expression of 883 proteins at a genome-wide false discovery rate <5%. In contrast to expression at the transcript level and complex diseases that are found to be mainly influenced by noncoding variants, we found protein expression level tends to be regulated by non-synonymous variants. We also provided evidence of 76 shared regulatory signals between gene expression and protein abundance. Mediation analysis revealed that for most (88%) of the colocalized genes, the expression levels of their corresponding proteins are regulated by *cis*-pQTLs via gene transcription. Using summary data-based Mendelian randomization analysis, we identified 4 proteins and 19 genes that are causally associated with schizophrenia. We further integrated multiple omics data with network analysis to prioritize candidate genes for schizophrenia risk loci. Collectively, our findings underscore the potential of proteome-wide linkage analysis in gaining mechanistic insights into the pathogenesis of psychiatric disorders.

## Introduction

Psychiatric disorders are complex polygenic diseases that are influenced by both genetic and environmental factors [1, 2]. Schizophrenia (SCZ) and bipolar disorder (BP) are two of the most prevalent psychiatric disorders, with 12-month prevalence of ~0.4−0.72% [3] and ~1.9% worldwide [4, 5], respectively. The two disorders share neurobiological alterations and genetic vulnerability [1, 6–9]. The Psychiatric Genomics Consortium (PGC) estimated a 68% genetic correlation between BP and SCZ using genome-wide single nucleotide polymorphisms (SNPs) [10]. The heritability of both disorders is very high, with 81% for SCZ [11, 12] and 85% for BP [13–16]. Psychiatric disorders impose a considerable economic burden on society due to the early age of onset, chronicity, and lack of efficient treatments or prevention strategies [17, 18]. Current treatments, such as antidepressants, antipsychotics, and neurostimulation, are only partially effective [19], and the development of better treatments is hindered by limited understanding of the underlying molecular mechanisms of psychiatric disorders.

Over the past decade, genome-wide association studies (GWAS) have successfully identified hundreds of genomic loci associated with psychiatric disorders [20–23]. However, we have little understanding of molecular mechanisms affecting the disorders for most of these genomic loci. Gene expression quantitative trait locus (i.e., eQTL) has been used to study the genetic regulation of molecular phenotypes to identify targets implicated in psychiatric disorders [24–26], and other endophenotypes (e.g., methylation and chromatin activity) are also used to understand the complex genetic basis of psychiatric disorders [26, 27]. Recently, multi-omic [28] and cell-type-specific data [29] were employed to dissect the molecular mechanisms underlying the disorders. Proteins are essential players in a diverse range of biological processes and changes in mRNA and protein levels are often not correlated [30, 31]. Protein expression is regulated at multiple levels, including transcriptional and post-transcriptional regulations that affect RNA stability, protein translation, and protein turnover and degradation. Each of these regulations can be influenced by genetic variation. However, the genetic landscape of proteome-wide regulation in psychiatric disorders remains largely unexplored.

Liquid chromatography coupled with tandem mass spectrometry (LC-MS/MS) technology has become a powerful platform for identifying and quantifying proteins [32]. Several attempts have recently been made to proteome-wide define genomic loci associated with protein expression in human cell lines [33], plasma [34, 35] and post-mortem brain tissues [36, 37] from Alzheimer’s disease (AD). The combination of proteomics and genetics studies has yielded valuable insights into how genetic variants are mechanistically linked to diseases [38]. However, little is known regarding the impact of genetic variants on psychiatric disorders by modulating protein expression in the human brain.

**Fig. 1:**
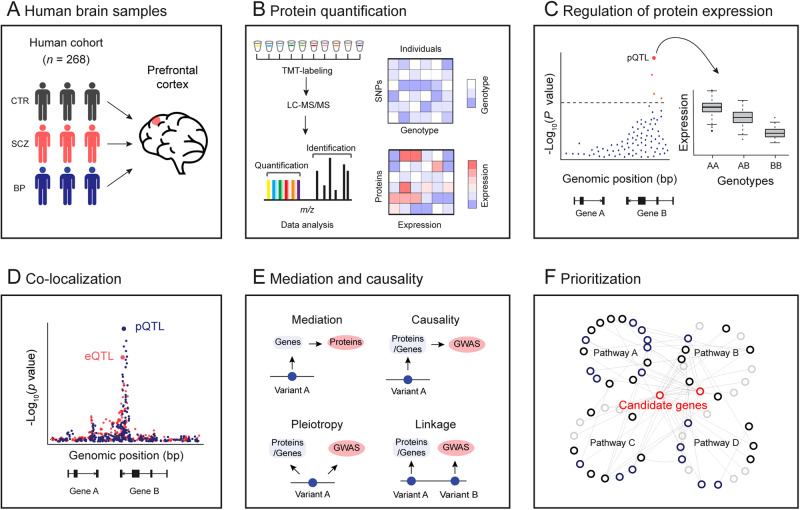
Schematic diagram showing the experimental design and analysis pipeline used in this study. **A** Postmortem brain samples from a human cohort with 268 participants were used, including 198 normal individuals (CTR), 45 patients with schizophrenia (SCZ), and 25 patients with bipolar (BP). **B** Deep brain proteome was profiled by 11-plex TMT-based proteomics, followed by extensive quality control and data analysis. Brain proteomic data and comparable genotype data were prepared for subsequent linkage analysis. **C** Genome-wide association analysis to identify genetic regulations of protein expression and gene expression. **D** Co-localization analysis to investigate the same variant underlying *cis*-eQTLs and *cis*-pQTLs. **E** Mediation analysis to identify transcript-dependent and -independent regulations and causality analysis to link eGenes and pGenes to SCZ GWAS loci. **F** Prioritization of proteins for SCZ GWAS loci.

To gain a better understanding of how genetic variation influences protein expression in the human brain and ultimately impacts psychiatric disorders, we perform a deep proteome and transcriptome profiling of the post-mortem frontal cortex of a human cohort (Fig. 1A, B), followed by genetic analysis to identify genomic loci associated with gene expression (i.e., eQTL) and protein expression (i.e., pQTL) (Fig. 1C) and colocalization analysis of pQTL and eQTL signals (Fig. 1D). To understand how these pQTLs and eQTLs contribute to the pathogenesis of psychiatric disorders, we further integrate pQTLs and eQTLs with GWAS loci to identify risk genes that are involved in the pathology of SCZ and BP (Fig. 1E). We finally integrate multi-omic bulk and single-cell transcriptomic data to prioritize risk genes/proteins for SCZ GWAS loci (Fig. 1F).

## Results

### Profiling and analysis of human brain proteome and transcriptome

**Fig. 2:**
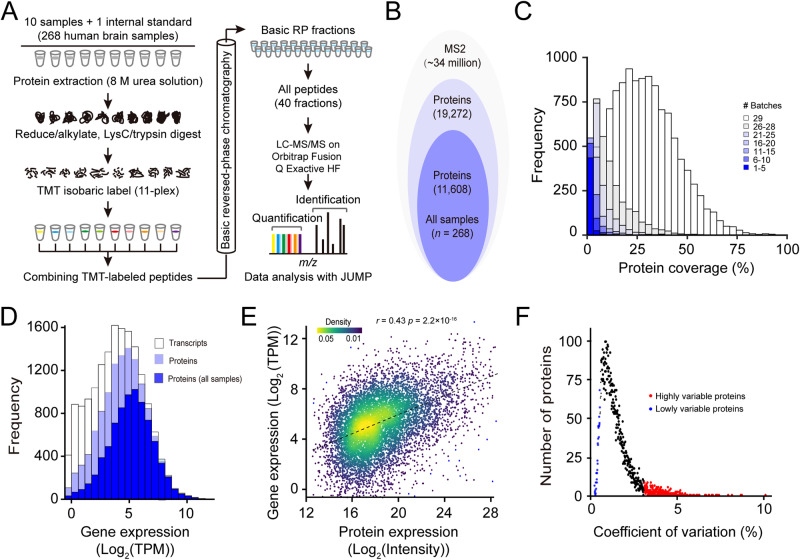
Deep profiling of human brain proteome. **A** Workflow of 11-plex TMT-based proteome analysis. A total of 10 samples and 1 internal standard (i.e., 10 pooled samples) were analyzed by LC/LC-MS/MS. MS raw data were analyzed using JUMP software. **B** Stacked Venn diagram showing the numbers of proteins identified in all 268 samples. **C** Histogram showing the coverage of quantified proteins across 29 batches of TMT experiments. **D** Histogram showing the coverage of proteomic data compared to RNA-seq data. The open bar represents the distribution of protein-coding genes detected by RNA-seq, the light blue bar indicates the distribution of protein-coding genes from proteomic data, and the navy bar indicates the distribution of protein-coding genes from no missing value proteomic data. Protein coverage is defined as the determination of whether a transcript is expressed in one or more samples. **E** Scatter plot showing a comparison of gene expression levels and protein abundance. Expression levels are averaged across all samples. **F** Distribution of coefficient of variation (CV) for all proteins across all samples.

To explore how genetic regulation of protein expression in the brain is implicated in psychiatric disorders, we generated deep proteomic data from the frontal cortex tissue of post-mortem human brains. These samples were analyzed by extensive fractionation (two-dimensional LC) and high-resolution, accurate-mass, tandem mass spectrometry (LC/LC-MS/MS) (Fig. 2A). We identified and quantified a total of 19,272 proteins (14,221 genes) in at least one sample at the protein FDR < 1% across 29 batches of 11-plex tandem mass tags (TMT) experiments. After extensive quality control measures [39], we focused on 11,608 proteins (8321 genes) from 268 samples, including 198 normal, 25 BP and 45 SCZ samples of high quality for the subsequent proteome-wide genetic regulation analysis (Fig. 2B, Table S1A, B; Table S2). The vast majority of proteins (78.07%; 15,045/19,272) were detected in more than 25 batches (Fig. 2C). To the best of our knowledge, this is the deepest human proteome data to date available for studying the genetic regulation of protein expression in the brain.

We next compared our proteomic to transcriptomic data generated from the matched 264 samples (Table S3). The quantified proteins across all samples cover ~70% of the range of mRNA expression detected by RNA-seq, indicating a deep coverage of our proteomic data (Fig. 2D). Those undetected proteins, whose corresponding RNAs also had low expression signals, are likely due to either not being translated into proteins in the brain or at concentration under the detection limit by the mass spectrometer. A moderate positive correlation (*r* = 0.43) was observed between expression levels of mRNAs and proteins (Fig. 2E), which is consistent with previous findings [30, 31, 33]. A subset of proteins showed high variability in expression (Fig. 2F), which were mainly enriched in functional terms related to extracellular matrix (ECM) organization, blood microparticle, plasma lipoprotein particle, and integrin binding (Supplementary Fig. 1A), whereas proteins with low variability were enriched in terms associated with the housekeeping functions, such as proteasome complex, regulation of mRNA stability, and regulation of cell cycle (Supplementary Fig. 1B).

### Human brain proteome and transcriptome reveal the genetic architecture of expression regulation

To characterize genetic variants influencing expression level of genes and proteins, we performed proteome-wide and transcriptome-wide association analyses. To increase statistical power and reduce false positives [40, 41], we removed variously measured and unmeasured confounding factors, such as experimental and technical batch effects. By using the probabilistic estimation of expression residuals (PEER) program [42], we captured 99% of the hidden variance in proteomic data with 13 controlling factors. Further correlation analysis indicated that the effects of various covariates on protein expression variation have been well-controlled (Supplementary Information; Supplementary Fig. 2A–D).

**Fig. 3:**
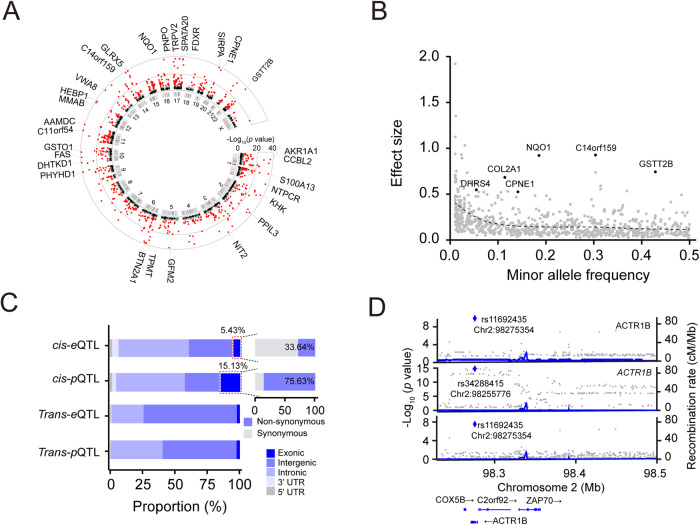
Genetic regulation of the brain proteome. **A** Circos plot showing genome-wide *cis*-pQTLs. Significant *cis*-pQTLs (*q* < 0.05) are highlighted in red color. **B** Scatter plot showing the relationship between minor allele frequency (MAF) and effect size of significant *cis*-pQTLs. SCZ risk genes with a large effect size (*β* > 0.5) are also labeled in the plot. **C** Stacked bar chart illustrating the proportions of each class of QTLs found in different genomic regions. **D** LocusZoom plot illustrating the colocalization of *cis*-eQTL, *cis*-pQTL, and GWAS locus.

We first performed genome-wide association analysis (Supplementary Fig. 3) of the expression levels of 11,608 proteins using the QTLtools program [43], identifying 788 *cis*-acting (or local acting) genomic loci (i.e., *cis*-pQTLs; within ±1 Mb from the transcriptional start site for each tested protein) that modulate the expression of 883 proteins (i.e., pGenes) at the genome-wide FDR < 5% using the Storey *q* value method (Fig. 3A; Table S4A). We will use the terms FDR and “*q*-value” interchangeably for the linkage analysis hereafter. Many significant *cis*-pQTLs (10.8%) are associated with more than one protein as opposed to 3.1% of the *cis*-eQTLs, suggesting that *cis*-pQTLs tend to be more pleiotropic than *cis*-eQTLs. Of these, 42 *cis*-pQTLs have a relatively large effect size (*β* > 0.5). We found that a trend for alleles with lower frequency has a stronger effect on *cis*-pQTLs (Fig. 3B). We also detected 256 *trans*-acting (or distal-acting) loci that regulate 511 proteins at the genome-wide FDR < 5% (Table S4B**;** *p* < 2.33 × 10^−11^; Bonferroni Correction), of which 19 (3.9%) loci harbor both *cis-* and *trans-*pQTLs.

We next performed genome-wide association analysis for gene expression (i.e., eQTL) in 416 frontal cortex samples that include nearly all of the samples (264/268; Table S1C, D; Table S3) used for proteome-wide analysis. Similarly, we removed hidden confounding factors for 17,160 expressed genes using the PEER program (Supplementary Information; Supplementary Fig. 4A–D). We identified 9791 significant *cis*-eQTLs that modulate expression levels of 9960 genes (i.e., eGenes) at the genome-wide FDR < 5% (Supplementary Fig. 5A; Table S5A), including 560 *cis*-eQTLs with large effect size (*β* > 0.5; Supplementary Fig. 5B). We also identified that expression levels of a total of 271 genes are regulated by 260 *trans*-eQTLs (Table S5B). A positional enrichment analysis showed that 56.28% (5605/9960) of significant *cis*-eQTLs cluster within 10 kb of the transcription starting site (TSS) of its target genes (Supplementary Fig. 5C). Interestingly, we observed that 15.13% of *cis*-pQTLs reside in exonic regions, with 75.63% of them being non-synonymous variants. This indicates that a total of 11.44% of *cis*-pQTLs can be attributed to non-synonymous variants. In contrast, only 5.43% of *cis*-eQTLs were detected in exonic regions, with 33.64% of which are non-synonymous variants, resulting in 1.83% of the total *cis*-eQTLs being non-synonymous exonic *cis*-eQTLs (Fig. 3C). This observation indicates that coding variants have a unique and significant impact on protein expression, which differs from the tendency of *ci*s-eQTLs to be located near the TSS region, as observed in this study and previous eQTL studies. The enrichment of *cis*-pQTLs in coding variants is consistent with recent proteome-wide association studies conducted in AD [44] and lymphoblastoid cell lines (LCLs) [45].

To understand the potential influence of Protein-Altering Variants (PAVs) on the regulation of protein expression levels, we utilized three well-established prediction algorithms, Combined Annotation Dependent Depletion (CADD) [46], Sorting Intolerant From Tolerant (SIFT) [47], and Polymorphism Phenotyping v2 (PolyPhen 2) [48] to predict PAVs with potentially deleterious effects. Out of 90 non-synonymous SNPs detected as *cis*-pQTLs, we identified a total of 63, 43, and 46 SNPs with deleterious effects on protein function as determined by CADD (score > 20), SIFT, and PolyPhen 2, respectively (Supplementary Fig. 6A; Table S6), with 38 SNPs being predicted to be deleterious by all three algorithms. As an example, a non-synonymous SNP at Chr2:98,275,354 in exon 4 in protein ACTR1B was predicted to be deleterious by all three tools (Supplementary Fig. 6B). ACTR1B had strong *cis*-eQTL (*p* = 8.49 × 10^−16^) and *cis*-pQTL (*p* = 2.83 × 10^−10^), and co-localized with a SCZ locus (Fig. 3D). The reference homozygous allele (G/G) decreases protein expression level compared to the homozygous alternative allele (A/A), (Supplementary Fig. 6C).

Among the 256 *trans*-pQTLs identified in this study, 11 were found to modulate the expression of more than five proteins (Supplementary Fig. 7). For example, a *trans*-QTL (*rs77546871*) in WW domain-containing oxidoreductase (WWOX) protein regulates the expression of 27 downstream proteins (Supplementary Fig. 7; blue lines). WWOX is also a significant *cis*-pGene (*p* = 9.19 × 10^−10^, *q* = 1.92 × 10^−4^). WWOX has been implicated in signaling pathways, such as regulating the central nervous system (CNS) development and neural differentiation [49], and dysfunction of this gene has been found to result in reduced GABA-ergic inhibitory interneuron numbers in mice [50]. GWA studies have also identified WWOX as a risk gene for common neurodegenerative conditions, such as SCZ [51], AD [52], and autism [53].

### Extensive colocalization between *cis*-pQTLs, *cis*-eQTLs, and GWAS signals

**Fig. 4:**
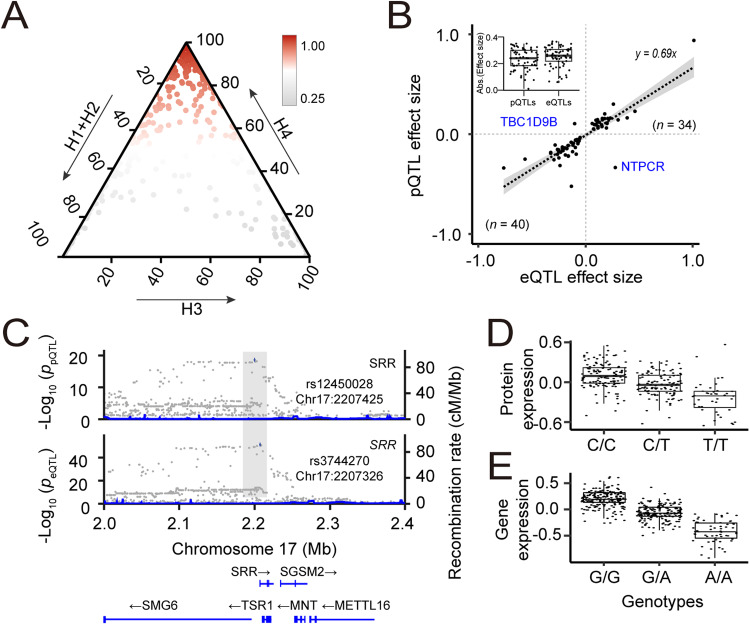
Co-localized QTLs modulating the expression levels of genes and proteins. **A** Ternary plot showing colocalization posterior probabilities of QTLs of gene and protein expression. We considered H_0_ + H_1_ + H_2_ as evidence for the lack of test power. H_0_: no causal variant, H_1_: causal variant for PD GWAS only, H_2_: causal variant for QTL only, H_3_: two distinct causal variants, H_4_: one common causal variant. **B** Scatter plot showing the distribution of effect sizes of 76 matched SNP-eQTLs and SNP-pQTLs colocalized pairs. **C** LocusZoom plot showing a colocalized QTL regulating SRR gene and protein expression. **D** Box plot showing normalized SRR protein expression and its *cis*-pQTL allele dosage. **E** Box plot showing normalized SRR gene expression and its *cis*-eQTL allele dosage.

To investigate the extent of colocalization between genetic variants associated with gene and protein expression and signals from GWAS of SCZ and BP, we first examined whether the association signals regulating gene and protein expression levels are driven by the same genetic variant. We performed a colocalization analysis for 883 pGenes and 9960 eGenes using the coloc program. The colocalization analysis estimates five posterior probabilities (PP0, PP1, PP2, PP3, and PP4) (see methods). We identified 660 pGenes with *cis*-eQTL signals within a 1 Mb distance (upstream or downstream). Among these, we found 386 pGenes (i.e., 346 genomic loci) with evidence of the colocalization (PP4 > 0.80; Fig. 4A; Table S7A). An over-representation analysis indicated a significant enrichment (Fisher exact test; *p* = 5.8 × 10^−3^) of colocalized *cis*-QTL signals.

Out of the 386 colocalized *cis*-QTL signals, 76 had matched SNP-eGene and SNP-pGene pairs. We examined the effect size of these matched pairs and observed a high consistency in the direction of effect size between eQTLs and pQTLs (Fig. 4B). The effect size of colocalized *cis*-pQTLs is slightly smaller compared to that of their corresponding *cis*-eQTLs (Fig. 4B inset), in agreement with the previous observation [45]. As an example, we illustrate serine racemase (SRR) protein to show the colocalization of *cis*-eQTL and *cis*-pQTL signals, which had a large posterior probability of colocalization (*PP*_*4*_ = 0.99). SRR was identified as a significant *cis*-pQTL (*p* = 2.64 × 10^−19^, *q* = 3.71 × 10^−13^), and a significant *cis*-eQTL (*p* = 4.88 × 10^−87^, *q* = 5.82 × 10^−75^) (Fig. 4C–E). SRR is a highly expressed protein in the brain acting as an endogenous ligand of N-methyl d-aspartate (NMDA) receptors. Disruption of the SRR protein was shown to reduce the function of NMDA receptors and is associated with susceptibility to SCZ [54].

We next conducted a colocalization analysis between *cis*-pQTLs/*cis*-eQTLs and GWAS loci. We found 12 *cis*-pQTLs colocalized with SCZ GWAS signals [55] and 2 *cis*-pQTLs colocalized with BP GWAS signals [56], (PP4 > 0.80; Supplementary Fig. 8A–C; Table S7B, C). Furthermore, we identified 65 and 21 *cis*-eQTLs colocalizing with SCZ and BP GWAS signals, respectively (PP4 > 0.80; Supplementary Fig. 8A, D, E; Table S7D, E). For example, we found angiotensin-converting enzyme (ACE) that had a strong colocalized signal between *cis*-eQTL (*p* = 2.20 × 10^−18^), *cis*-pQTL (*p* = 1.73 × 10^−9^), and SCZ GWAS loci (Supplementary Fig. 8F).

### Mediation analysis elucidates the regulation of protein expression

**Fig. 5:**
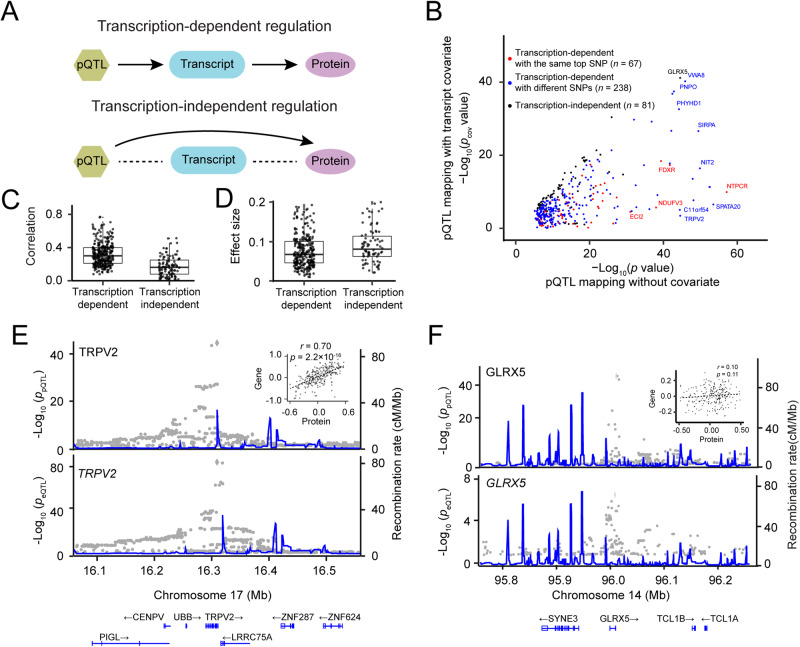
Genetic regulation of protein expression mediated by mRNA. **A** Two mediation models of protein expression: transcription-dependent protein regulation and transcription-independent protein regulation. **B** Scatter plot showing negative log-transformed *p* values of *cis*-pQTL before and after conditioning on mRNA. **C** Box and whisker plot showing Pearson correlation coefficient between expression levels of proteins and transcripts in both transcript-mediated and transcript-independent groups. The plot shows the mean (horizontal lines), 5^th^–95^th^ percentile values (boxes), and SEM (whiskers). **D** Box and whisker plot showing effect sizes of transcription-dependent and transcription-independent regulations. **E** An example of transcription-dependent regulation is exemplified by TRPV2. LocusZoom plots show a significant localization of *cis*-pQTL (top) and *cis*-eQTL (bottom). The inset shows the scatter plot of a high correlation (*r* = 0.70) between the expression of gene and protein. **F** Transcription-independent regulation is exemplified by GLRX5. LocusZoom plots show a significant *cis*-pQTL but not a *cis*-eQTL. The inset shows the scatter plot of a low correlation (*r* = 0.10) of GLRX5 expression levels between gene and protein.

To investigate whether protein expression is dependently regulated by its *cis*-pQTL through the corresponding mRNA transcription [57] (Fig. 5A), we performed a conditional mapping for the 386 colocalized pGenes using the corresponding gene expression as a co-variate (Supplementary Fig. 9A). We observed that the expression level of a majority of pGenes (305/386, 79%) is regulated by eGenes (Fig. 5B; Table S8), suggesting that these protein regulations were largely regulated through transcriptional mechanisms (i.e., transcription-dependent regulation). In addition, a substantial proportion of matched SNP-pGenes and SNP-eGenes (67/76; 88%) exhibited transcription-dependent regulation.

This transcription-dependent protein regulation was supported by a modest correlation (*r* = 0.34) between transcripts and proteins (Fig. 5C), which is significantly higher (*p*  < 2.2 × 10^−16^) than those transcription-independent pGenes (*r* = 0.14). However, the effect size of transcription-mediated pGenes was significantly lower as compared to that of transcription-independent pGenes (Fig. 5D; 0.11 vs 0.18; *p* = 6.5 × 10^−6^), suggesting direct genetic effects on protein abundance tend to be stronger than the mediation effects. As expected, the vast majority of transcription-dependent pQTLs are found to be in the genomic regulatory regions (Supplementary Fig. 9B).

To illustrate transcription-dependent regulation, we highlighted an example of transient receptor potential cation channel subfamily V member 2 (TRPV2), an ion channel protein. TRPV2 showed a significant *cis*-pQTL (*p* = 2.93 × 10^−45^, *q* value = 2.11 × 10^−27^), but the signal was abolished after conditioning on gene expression as a co-variate. TRPV2 level at the gene level is also regulated by a significant *cis*-eQTL (*p* = 3.70 × 10^−97^, *q* value = 2.66 × 10^−58^) (Fig. 5E). The transcript and protein expression levels of TRPV2 are highly correlated (Fig. 5E; inset). In the case of the transcription-independent regulation, we found a significant *cis*-pQTL that regulates GLRX5 protein abundance independently of its transcription (Fig. 5F). As expected, there is a lack of correlation between protein and transcript abundance (Fig. 5F; inset).

### Causal contribution of *cis*-pGenes and *cis*-eGenes to psychiatric disorders

**Fig. 6:**
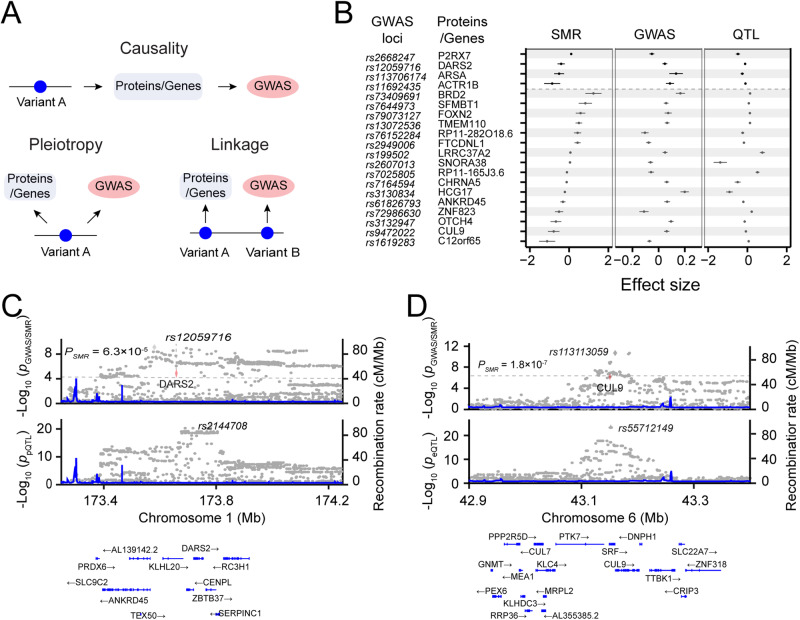
Causal relationship between pGenes and SCZ. **A** Schematic diagram showing three putative mechanistic controls of a QTL: causality, pleiotropy, and genetic linkage. **B** Forest plots showing effect sizes of 4 and 19 SCZ GWAS loci causally controlled by pGenes and eGenes, respectively. The causality relationship was estimated by the SMR/HEIDI method. Center values mark effect size point estimates, error bars the 95% confidence intervals. **C** LocusZoom plot showing an example of an SCZ GWAS is controlled by a *cis*-pQTL. **D** LocusZoom plot showing an example of an SCZ GWAS is controlled by a *cis*-eQTL.

We next sought to identify genomic loci associated with psychiatric disorders through genetic effects on gene and protein expression. We evaluated 287 SCZ genomic loci identified by a meta-analysis of recent published data from the Psychiatric Genomics Consortium (PGC) [55]. We used summary-based Mendelian randomization (SMR) analysis coupled with the heterogeneity independent instruments (HEIDI) test [58] (Fig. 6A), identifying 4 pGenes that passed both the HEIDI heterogeneity test (*P*_HEIDI_ > 0.05) and the SMR significance threshold of *P*_SMR_ < 6.3 × 10^−5^ (0.05/790; *p* = 0.05 corrected by the total number of pGenes) (Fig. 6B; Table S9A). We also detected 19 eGenes that passed *P*_HEIDI_ > 0.05 and *P*_SMR_ < 5.3 × 10^−6^ (0.05/9,495; corrected by 9,495 eGenes) (Fig. 6B; Table S9B). Among these pGenes and eGenes with significant *cis*-pQTL and *cis*-eQTL, 2 pGenes and 13 eGenes were also prioritized for the SCZ GWAS loci. Note that the SMR analysis cannot distinguish causality from pleiotropy. In addition, one protein (BTN2A1) and 21 genes showed *P*_HEIDI_ < 0.05 from the HEIDI test, suggesting that expression and GWAS are likely to be driven by different variants in the same linkage disequilibrium block. We also evaluated 64 genomic loci recently identified by BP GWAS meta-analysis [56], identifying 4 causal/pleoitropic eGenes (*P*_SMR_ < 5.3 × 10^−6^; Supplementary Fig. 10; Table S9C).

An example of the causality effect of pGene on SCZ is DARS2, a mitochondrial aspartyl-tRNA synthetase. The SMR analysis detected a significant association between DARS2 protein expression and SCZ (*P*_SMR_ = 1.66 × 10^−5^ and *P*_HEIDI_ = 0.16). DARS2 is a significant pGene (*p* = 5.88 × 10^−21^, *q* = 4.93 × 10^−15^) (Fig. 6C), which is highly expressed in the brain and has been identified as the strongest causal gene of SCZ in an independent GWAS12. As an example of a significant causal association between eGenes and SCZ, CUL9 (cullin-9) exhibited a significant association between gene expression and SCZ, with *P*_SMR_ = 1.78 × 10^−7^ and *P*_HEIDI_ = 0.11 (Fig. 6D; Table S9B). CUL9 is a parkin-like ubiquitin ligase that has been prioritized as a candidate gene for an SCZ GWAS locus [55].

### Integrative analysis prioritizes proteins for psychiatric disorders

Previous studies have shown that molecular QTLs (e.g., eQTLs, methylation QTLs (mQTLs), and pQTLs) tend to influence complex diseases [59], and they can be harnessed to prioritize risk genes for GWAS loci [60]. Although it is currently difficult to pinpoint causal genes at GWAS loci, prioritized genes/proteins could be plausible candidates underlying the GWAS associations. In this study, we attempt to establish a framework to systematically prioritize risk genes for 313 significant SCZ GWAS loci (*p* < 5 × 10^−8^) and 311 suggestive loci (5 × 10^−8^ < *p*  < 1 × 10^−6^) with small effect size [55].

**Fig. 7:**
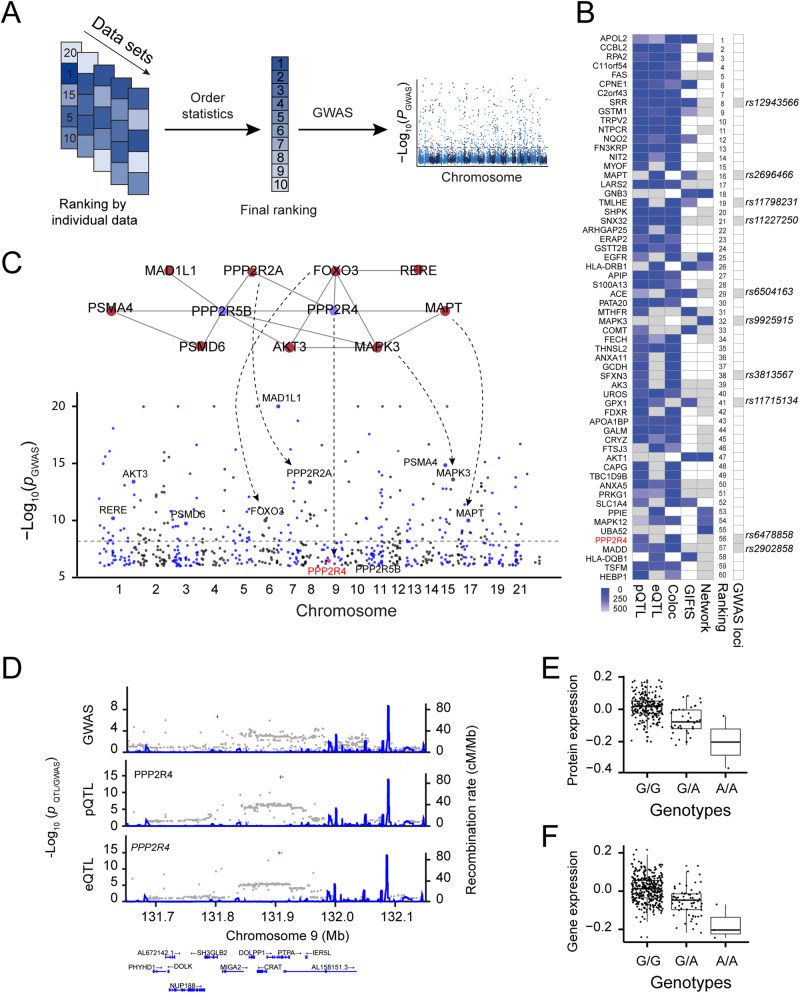
Prioritization of candidate genes for SCZ GWAS loci by integrating multiple data sets. **A** Schematic diagram of candidate gene prioritization using order statistics. **B** Heatmap showing the top 60 proteins ranked by combining five data sets. The missing values are indicated by white boxes. **C** Network-based reprioritizing candidate genes for SCZ GWAS associations with small effect. Sub-network (top) was derived from the STRING PPI network. Significant GWAS risk genes are indicated by red nodes, whereas candidate genes (PPP2R4 and PPP2R5B) for suggestive GWAS loci are indicated by blue nodes. **D** LocusZoom plot showing a SCZ GWAS locus (*rs6478858*), a colocalized QTL regulating PPP2R4 gene and protein expression levels. **E** Box plot showing normalized SRR protein expression and its *cis*-pQTL allele dosage. **F** Box plot showing normalized SRR gene expression and its *cis*-eQTL allele dosage.

We sought to combine multiple data sets to prioritize genes/proteins for GWAS loci using order statistics (Fig. 7A). Five data sets were included for the prioritization, including pGenes ranked by *cis*-pQTL nominal *p* values, eGenes ranked by *cis*-eQTL nominal *p* values, co-localization between *cis*-pQTLs and *cis*-eQTLs ranked by PP_4_ values, and disease relevance score with SCZ by the GeneCards database, and connectivity score ranked by the number of downstream SCZ risk genes in protein-protein interaction (PPI) network (see Methods; Supplementary Fig. 11A). To derive the PPI network connectivity score, we first extracted high-confidence PPI with a score ≥ 700 (mean score: 295, range: 150–999) and kept those nodes with *cis*-pGenes or *cis*-eGenes (Supplementary Fig. 11B), yielding an SCZ network with 2011 nodes and 3118 protein-protein interactions (Supplementary Fig. 11B). We used order statistics to generate a final ranking score, followed by identifying candidate genes for GWAS loci. To further assess the cell-type-specific differential expression of these ranked proteins, we also downloaded single-cell transcriptomic data generated from 48 post-mortem human prefrontal cortex samples, including 24 schizophrenia cases and 24 controls [61] and mapped differential expression genes between SCZ and controls and expression abundance of 20 cell types to our ranked proteins (Table S10). The final ranking result revealed that among the top-ranked 60 proteins, 8 candidate genes were from the 313 significant PGC SCZ GWAS loci (Fig. 7B; Table S10) and 2 additional candidate genes were SCZ GWAS loci from other studies. Single-cell transcriptomic data [61] support that 30 out of the top 60 proteins showed excitatory neuron cell-state (Ex-SZTR, which are enriched for differentially-expressed genes and significantly more prevalent in schizophrenia than in control individuals, but preferentially found in schizophrenia individuals with non-schizophrenia transcriptional signatures across all other cell types in addition to excitatory neuron) (Supplementary Fig. 12).

A major challenge in GWAS is unable to detect loci with a small effect due to low statistical power [57]. We next leveraged our prioritized proteins to identify candidate risk genes for 290 suggestive GWAS loci (5 × 10^−8^ < *p* < 1 × 10^−6^) with a smaller effect size. We found 2 out of the top 60 ranked genes in suggestive GWAS loci (Fig. 7B). For example, PPP2R4 is prioritized as a candidate gene for a GWAS locus *rs6478858*. This is also supported by evidence that PPP2R4 is functionally associated with SCZ risk genes that include MAPT, PPP2R2A, FOXO3, AKT3, RERE, RSMO6, PSMA4, and MAD1L1 (Fig. 7C). PPP2R4 showed a colocalized significant *cis*-pQTL (*p* = 2.70 × 10^−15^, *q* value = 1.58 × 10^−8^) and *cis*-eQTL (*p* = 8.92 × 10^−16^, *q* = 2.29 × 10^−12^) (Fig. 7D). The reference homozygous allele (G/G) increases protein and gene expression levels by 1.16-fold and 1.14-fold compared to the homozygous alternative allele (A/A), respectively (Fig. 7E, F). Single-cell transcriptomic data showed that PPP2R4 decreases the expression level in microglia and is differentially expressed in SCZ compared to control samples (Supplementary Fig. 12). These results suggest that our protein prioritization provides a potential strategy for identifying candidate genes in GWAS loci with small effects.

## Discussion

In this study, we performed proteome-wide and transcriptome-wide association studies of post-mortem brain tissue from a human cohort of controls and patients with psychiatric disorders. We characterized the genetic architecture of human gene and protein regulation by discovering 9791 *cis*-eQTLs and 788 *cis*-pQTLs that regulate gene and protein expression, respectively. Our causality analysis highlighted eGenes and pGenes that are functionally implicated in psychiatric disorders. Prioritization analysis further revealed proteins as candidate risk genes for SCZ GWAS loci. Taken together, the findings of this study increase our understanding of the genetic regulation of gene and protein expression in the human brain and shed light on the underlying molecular mechanisms involved in psychiatric disorders.

One of the strengths of this study was that we comprehensively defined the landscape of genetic regulation of protein expression by quantifying 11,608 unique proteins across all 268 human brain samples. We quantified a total of 19,272 unique proteins from at least one batch of TMT-based proteomic experiments (Fig. 2B). To the best of our knowledge, this is the deepest human brain proteome reported to date for such a large human cohort. Compared to a recent large-scale human brain proteomic study [30], our proteomic data detected ~43.08% (11,608 vs. 8356) more unique proteins. This deep proteomic data provides an opportunity to comprehensively evaluate genetic loci regulating protein expression even with lowly expressed proteins, which otherwise remain undiscovered or poorly characterized with shallow proteomic data.

The availability of transcriptome and proteome of the brain tissue from the same human cohort in this study provided an excellent opportunity to investigate the commonalities and disparities in gene and protein expression regulations. We provided evidence that the vast majority (321/386) of colocalized *cis*-eQTLs and *cis*-pQTLs exhibited the same regulatory direction (Supplementary Fig. 13A), but the effect size of *cis*-pQTLs is generally smaller than that of *cis*-eQTLs (Supplementary Fig. 13B), indicating that their potential effects on downstream phenotypes were often attenuated or buffered [25, 46]. We also identified two *cis*-QTLs that showed an inconsistent direction of effect on eGenes and pGenes (i.e., TBC1D9B and NTPCR) (Fig. 4B; Supplementary Fig. 13C, D). NTPCR had strong significant *cis*-eQTL (*p* = 4.12 × 10^−90^) and *cis*-pQTL (*p* = 7.72 × 10^−58^), but showed a negative correlation between gene and protein expression levels, suggesting a likelihood of the pleiotropic effect of the variant. On the other hand, despite exhibiting significant *cis*-eQTL (*p* = 9.59 × 10^−25^) and *cis*-pQTL (*p* = 1.11 × 10^−18^), TBC1D9B showed no correlation (*r* = −0.10; *p* = 0.09) between gene and protein expression levels (Supplementary Fig. 13D), suggesting a possibility of false QTL signals.

Another advantage of measuring gene and protein expression in the same tissue from the matched samples is that it allows us to investigate the mediation of protein regulation. Although protein expression often correlates poorly with transcript levels, we observed that most of the pGenes (274/386) were colocalized with eQTLs signals, and the expression level of most of these colocalized pGenes are modulated by gene transcription. This observation is consistent with previous reports that the vast majority of genetic variants controlling gene expression also influence protein abundance [62]. In addition, we also observed about some of the pGenes are regulated in a transcription-independent manner. For these pGenes, post-transcriptional regulation often buffers differences in the genetic regulation of protein abundance from mRNA levels [24].

In the present study, we identified 883 pGenes, a number substantially lower than the number of 9960 eGenes detected. A plausible possibility for this observation is the relatively smaller sample size used for detecting pQTLs compared to eQTL detection. Moreover, we posit that the buffering of genetic variation at the protein level could account for this difference [44, 62, 63]. The most widely recognized mechanism that buffers genetic variation is redundancy. The redundancy may arise due to various post-transcriptional and post-translational regulations that can modulate protein expression levels independently of mRNA levels. For example, the human genome has more proteins than genes. The family of protein members can substitute for one another when inappropriately expressed.

The detection of pQTLs can be influenced by population structure, which introduces confounding factors. To assess the impact of population structure on pQTL detection in this study, we compared *cis*-pQTLs detected by QTLtools without considering population structure with those detected by the GEMMA program with taking into consideration relatedness. We found 88% (781/883) of significant pGenes identified by both QTLtools and GEMMA analyses (Supplementary Fig. 14). This result suggests that the population structure has a marginal impact on the detection of pQTLs. The GEMMA method with population structure detected more significant *cis*-pQTLs, suggesting that incorporating population structure in the QTL mapping can slightly improve statistical power.

The low expression level may produce false positive eQTLs in which the major allele was associated with lower gene expression levels. In this study, we used genes with transcripts per million reads (TPM) > 0.1 in at least 25% of samples. To evaluate the impact of low expression on eQTL detection, we first explored the distribution of positive *cis*-eQTLs across different gene expression levels. Our analysis revealed a consistent rate of positive *cis*-eQTLs throughout the expression spectrum (Supplementary Fig. 15A). We observed a slightly lower rate of positive *cis*-eQTLs for genes with low expression levels (TPM < 1; Log_2_(TPM) < 0). The analysis supports that there is no inflation in the subset of genes with low expression. To further validate our findings, we randomly selected six significant *cis*-eQTLs regulating genes displaying low expression levels (TPM < 1; Log_2_ (TPM) < 0; Supplementary Fig. 15B). Our manual examination further confirmed that these *cis*-eQTLs indeed displayed true eQTL signals, as evidenced by both Manhattan and box plots (Supplementary Fig. 15C). We conducted further investigation into 33 significant *cis*-eQTLs (*q* < 0.05) that regulate genes with low expression (Log_2_(TPM) < 0) by only two homozygote genotypes. Our analysis revealed that the majority of these genes showed positive signals, as illustrated by two examples (Supplementary Fig. 15D). However, it is worth noting that one gene had a  borderline significant *cis*-eQTL (*q* = 4.06 × 10^−5^, Supplementary Fig. 15E). Our analyses suggest that low expression could potentially exert a marginal influence on eQTL detection.

While we measured protein expression in the frontal cortex, a human brain region is still highly heterogeneous, containing different cell types [64]. Recent advances in single-cell transcriptomics have demonstrated the feasibility of identifying cell-type-specific eQTLs [64], which allows us to characterize the cellular specificity of genetic regulation of gene expression. In this study, we might be able to define cell-type-specific pQTLs by computationally deconvoluting sample-wise cell-type-specific expression from our bulk proteomic data. For example, CIBERSORTx [65] was developed for deriving a signature matrix and sample-wise deconvolution from the single-cell transcriptomic data. Although single-cell proteomics is still in its infancy, several promising technologies are being explored, such as nanoTOPS and SCoPE-MS [66]. For example, nanoTOPS is capable of identifying ~2000 proteins at 100-μm spatial resolution [67]. With the advent of single-cell proteomics technology, we will be able to define the genetic regulation of protein expression at the cellular level.

In summary, we provided a comprehensive resource on protein expression in the brain across a human cohort with control individuals and patients with psychiatric disorders. We defined a landscape of the genetic regulation of protein expression in the brain, highlighting a large set of variants and targets involved in molecular mechanisms underlying psychiatric disorders. We developed a framework to investigate the mediation of the protein expression and the causal link of eQTLs/pQTLs to genomic loci detected in the larger meta-GWAS study. We believe that integrating GWAS and genetic regulation of protein expression provides a new avenue for identifying novel risk genes for GWAS loci, thereby providing important insights into the pathogenesis of psychiatric disorders.

## Methods

### Human postmortem brain tissue

For proteome profiling, a total of 268 well-characterized postmortem human brain samples (165 males, 103 females) from the Stanley Medical Research Institute (SMRI) and Banner Sun Health Research Institute (BSHRI) were used for this study. These samples were collected from 198 neurotypical controls, 45 individuals with SCZ, and 25 individuals with BP (Table S1A, B). The samples include 262 Caucasians, 1 Hispanic American, 3 Asian American, and 2 Unknown. For transcriptome profiling, RNA-Seq data from 416 samples (262 males and 154 females). More detailed information about the specimens is provided in Table S1C, D.

### Brain tissue lysis and protein quantification

Frozen tissues from the frontal cortex (**BA46**) were obtained from controls and patients with SCZ and BP. The tissues were weighed and homogenized in lysis buffer (50 mM HEPES, pH 8.5, 8 M urea, and 0.5% sodium deoxycholate, 100 µl buffer per 10 mg tissue) with a 1×PhosSTOP phosphatase inhibitor cocktail (Sigma-Aldrich). The total protein concentration of each sample was measured by the BCA Protein Assay Kit (Thermo Fisher Scientific), and confirmed by Coomassie-stained short SDS gels.

### Protein digestion and TMT labeling

We used our previously optimized protocol [67, 68] for this analysis. In brief, quantified protein samples (~0.3 mg in the lysis buffer with 8 M urea) were proteolyzed with Lys-C (Wako, 1:100 w/w) at room temperature for 2 h, diluted 4-fold to reduce urea to 2 M, and digested by trypsin (Promega, 1:50 w/w) at room temperature overnight. The digestion was terminated by the addition of 1% trifluoroacetic acid, followed by centrifugation. The supernatant was desalted with Sep-Pak C18 cartridge (Waters), and then dried by speedvac. Each sample was resuspended in 50 mM HEPES, pH 8.5, labeled with 11-plex TMT reagents, mixed equally, and desalted again for subsequent fractionation. We used 0.1 mg protein per sample. A total of 29 batches of 11-plex TMT experiments were performed.

### Extensive two-dimensional LC/LC-MS/MS

The pooled TMT labeled samples were fractionated using offline basic pH reversed-phase chromatography (HPLC), and followed by acidic pH reverse phase LC-MS/MS analysis [68, 69]. For the offline basic HPLC, we generated 40 concatenated fractions for each batch. We performed the offline LC run (~3 h gradient) on an XBridge C18 column (3.5 μm particle size, 4.6 mm × 25 cm, Waters; buffer A: 10 mM ammonium formate, pH 8.0; buffer B: 95% acetonitrile, 10 mM ammonium formate, pH 8.0) [70]. For the acidic pH LC-MS/MS analysis, each fraction was run sequentially on a column (75 µm x 15−30 cm, 1.9 µm C18 resin, 65 °C to reduce backpressure) interfaced with an Orbitrap Fusion and Q Exactive HF MS (Thermo Fisher). Peptides were eluted by a 1.5−2 h gradient (buffer A: 0.2% formic acid, 5% DMSO; buffer B: buffer A plus 65% acetonitrile). MS settings included MS1 scans (60,000 resolution, 1 × 10^6^ AGC and 100 ms maximal ion time) and 20 data-dependent MS2 scans (410-1600 *m/z*, 60,000 resolution, 1 × 10^5^ AGC, ~105 ms maximal ion time, HCD, 38% normalized collision energy, 1.0 *m/z* isolation window with 0.2 *m/z* offset, and ~15 s dynamic exclusion).

### Identification of proteins by database search with JUMP software

We performed peptide identification with the JUMP search engine to improve the sensitivity and specificity [71]. JUMP searched MS/MS raw data against a composite target/decoy database [72] to evaluate FDR. The target human protein sequences (83,955 entries) were downloaded from the UniProt database. The decoy database was generated by reversing to generate a decoy database that was concatenated to the target database. FDR was estimated by the ratio of the number of decoy matches and the number of target matches. Major parameters included precursor and product ion mass tolerance ( ± 15 ppm), full trypticity, static mass shift for the TMT tags (+229.16293) and carbamidomethyl modification of 57.02146 on cysteine, dynamic mass shift for Met oxidation (+15.99491), maximal missed cleavage (*n* = 2), and maximal modification sites (*n* = 3). Putative PSMs were filtered by mass accuracy and then grouped by precursor ion charge state and filtered by JUMP-based matching scores (Jscore and ΔJn) to reduce FDR below 1% for proteins during the whole proteome analysis. If one peptide could be generated from multiple homologous proteins, based on the rule of parsimony, the peptide was assigned to the canonical protein form in the manually curated Swiss-Prot database. PSM-, peptide- and protein-level FDR were controlled using the target-decoy strategy [73]. Target and decoy spectral matches were distinguished from one another using linear discriminant analysis (LDA) based on several different parameters including Jscore, ΔJscore, precursor mass error, and charge state. The linear discriminant model was trained for individual LC-MS analyses using peptide matches to forward and reversed peptide sequences as positive and negative training data. Similar approaches have been published previously using different sets of features or different classifiers [74, 75]. After each was scored, sequences shorter than seven amino acids were discarded and peptide spectral matches were sorted by discriminant score and filtered to a 1% FDR as indicated by the number of decoy sequences in the filtered data set. PSMs with low confidence (Jscore < 50 for one-hit-wonder) were manually verified (Table S11).

### Protein quantification by JUMP software suite

Protein quantification was carried out using the following steps [76]. We first extracted the TMT reporter ion intensities of each PSM and corrected the raw intensities based on the isotopic distribution of each labeling reagent. We discarded PSMs with low intensities (i.e., the minimum intensity of 1000 and median intensity of 5000). After normalizing abundance with the trimmed median intensity of all PSMs, we calculated the mean-centered intensities across samples (e.g., relative intensities between each sample and the mean) and summarized protein relative intensities by averaging related PSMs. Finally, we derived protein absolute intensities by multiplying the relative intensities by the grand mean of the three most highly abundant PSMs. Log_2_-transformed data were used for the subsequent PEER factor analysis [42]. To determine the unique proteins, we considered only one canonical version if the identified peptides were shared by multiple isoforms. However, if an isoform was identified by its distinct peptides, it was also included in the list of unique proteins

### Genotypic data

We generated genotypic data from three different sources, namely Affymetrix, PsychcChip, and whole-genome sequencing. For Affymetrix and PsychcChip, we followed best practices for genotype calling and conducted thorough quality control checks to ensure high-quality data. In addition, we applied the GATK Haplotype caller to align the reads from whole-genome sequencing to the human reference genome and called the variants. We also used the BEAGLE method to refine the genotypes by inferring haplotypes and missing genotype data from the 1000G-EUR reference panel.

The genotypic data from each platform were imputed using Minimac3 and the Haplotype Reference Consortium (HRC) panel after standard quality control. After imputation, we filtered genotypes using R^2^ > 0.3 and HWE < 10^−6^ to obtain high-quality imputation data. We also corrected the identity of samples using our software, DRAMS. To generate the final genotypic data, we combined genotypes from the three platforms, set genotypes that did not match to missing values, and filtered genotypes by using missing rate <0.4 and minor allele frequency (MAF) < 0.01. We then re-imputed the missing values with the BEAGLE method and removed genotypes with HWE < 10^−6^ and genotypes within the ENCODE blacklist [77].

### Transcriptome profiling by RNA-seq

We used different RNA preparation techniques for human brain samples from the SMRI and the BSHRI collections. For SMRI brain samples, total RNA was isolated for SMRI samples through organic extraction. Briefly, approximately 50−60 mg of frontal cortex (**BA9 or BA46**) was homogenized by polytron probe in Trizol. Total RNA was precipitated with isopropanol at room temperature, pelleted, washed with 75% ethanol, and resuspended in DEPC treated water. Quantification was performed by obtaining OD at A260, and quality assayed by agarose gel electrophoresis. For BSHRI brain samples, total RNA was mixed with ethanol and applied to a miRNeasy mini-column. Columns were treated with the RNase-free DNase digestion set (Qiagen), then washed with the appropriate miRNeasy mini kit buffers. Total RNA was eluted with RNase-free water. All total RNA samples that passed QC for library generation had a concentration of ≥100 ng/uL, assayed by the Qubit 2.0 RNA BR Assay or Xpose, and a RIN score ≥5.5, assayed by the Bioanalyzer RNA 6000 Nano assay kit. Libraries were sequenced on the HiSeq4000 (Illumina).

### RNA-seq data analysis

All FASTQ files were trimmed for adapter sequence and low base call quality (Phred score <30 at ends) using cutadapt (v1.12) and then aligned to the GRCH37 (i.e., hg19) reference genome with STAR (2.4.2a) [78], using GENCODE gene annotations. BAM files were sorted using samtools (v1.3) [79]. Gene expression levels were quantified using RSEM (v1.2.29) [80]. Genes were filtered to include only those on autosomes longer than 250 base pairs with transcripts per million reads (TPM) > 0.1 in at least 25% of samples, removing immunoglobulin biotypes. Count-level quantifications were corrected for library size by using trimmed mean of M-values (TMM) normalization and were log_2_ transformed.

### PEER factor analysis

We employed the Probabilistic Estimation of Expression Residuals (PEER) method [42] to remove hidden batch and other confounding effects for both transcriptomic and proteomic data. A total of 30 and 13 covariate factors were identified in transcriptomic and proteomic data, respectively. These covariant factors captured ~99% of the total variance in both transcriptomic and proteomic data. We used the inverse normal-transformed PEER-processed residuals for downstream association analyses.

### Association analysis

We performed eQTL/pQTL mapping for both gene and protein expression using the QTLtools program (Version 1.2) [43] with the permutation number of 10,000. The top variant was selected as the QTL for the protein/gene. eQTLs/pQTLs were defined as *cis* (local) if the QTL was within 1 Mb on either side of the TSS, whereas eQTLs/pQTLs were defined as *trans* (distal) if the peak association was at least 5 Mb outside of the exon boundaries. We used beta distribution-adjusted empirical *p* values to estimate the *q* value by using the QVALUE R package. Significant *cis*-eQTLs and *cis*-pQTLs were controlled by the *q* value < 5%. Due to the large number of analyses for calculating *trans*-eQTL and *trans*-pQTL, we used the conservative Bonferroni-corrected *p* value of 0.05 (0.05/(number of genotypes x number of proteins) = 0.05/(8,101,465 × 11,608) = 5.3 × 10^−13^). A positive effect means the increase in expression level in the presence of the reference allele, whereas the negative effect indicates the decrease of the expression level in the presence of the alternative allele. To consider population structure in the pQTL mapping analysis, we performed the QTL analysis by the GEMMA software [81] that accounts for the population structure. Population structure were determined by the PLINK software [82].

### Functional annotation of QTLs

ANNOVAR [83] was used to functionally annotate the leading SNP of a QTL. RefSeq from the UCSC genome browser database was used to annotate SNPs. The functional consequence (synonymous, non-synonymous) of coding SNP was also determined.

### Co-localization analysis

We used the coloc R package [84] to analyze the colocalization between *cis*-eQTls and *cis*-pQTLs. A window size of 500 kb on either side of the pQTL was used. The coloc program uses a Bayesian model to determine posterior probabilities for five mutually exclusive hypotheses: no association of any variant in the region with either *cis*-pQTL and *cis*-eQTL (H_0_); association with *cis*-pQTL but not *cis*-eQTL (H_1_), association with *cis*-eQTL but not *cis*-pQTL (H_2_), two different QTLs (H_3_); and a shared QTL for both gene and protein expression (H_4_). These hypotheses were tested to produce the posterior probabilities, PP_*i*_ (*i* ∈ [0,4]). We consider PP_4_ > 0.5 to be significant evidence of colocalization.

### Causal/pleiotropic analysis of the effect of pGenes/eGenes on SCZ GWAS

We applied SMR [58] to test the causal/pleiotropic effect between genes/proteins and diseases using summary statistics from GWAS. In this study, SMR used SCZ GWAS loci as instrument variables and gene/protein expression levels as exposure to test whether the causal effect of a specific variant on the SCZ GWAS signal acts via a specific gene/protein. The SCZ GWAS loci were downloaded from the recently published Psychiatric Genomics Consortium (PGC) [55]. SMR performed the HEIDI (heterogeneity in dependent instruments) test to exclude the GWAS and pQTLs/eQTLs caused by genetic linkage. The HEIDI threshold (*P*_HEIDI_ > 0.05) and the SMR FDR-corrected threshold (adjusted *P*_SMR_ < 0.05) were used.

### Mediation analysis

The mediation analysis was performed to identify eGenes that are likely to be a causal mediator between the pQTL and the protein expression it regulates. We implemented this analysis with Perl language based on the conditional mapping function provided in the QTLtools. If the expression level of a protein is regulated by its *cis*-pQTL via the corresponding eGene as a mediator, the *p* value in the conditional pQTL mapping model (transcript as a covariate) should significantly decrease or abolish the pQTL effect. To assess whether the *p* value significantly drops for a given mediator on a *cis*-pQTL, a null distribution of *p* values was estimated by randomly permuting sample labeling of the eGene. For each protein, this analysis produces 1 *cis*- nominal *p* value and 1000 permutated nominal *p* values. The combined *p* values are then converted into z-scores. We consider a potential causal mediator with a z-score ≤ −4.26 (*p* = 1 × 10^−5^ significance level; 0.01/1000 multiple correction tests).

### PPI network of SCZ risk genes

GWASs have identified hundreds of GWAS loci associated with SCZ and BP. To manually curate a catalog of SCZ and BP risk genes, we extracted a total of 971 risk genes in GWAS loci reported by a list of 9 papers (Table S12). Detailed information about the studied subjects, diagnosis, genotyping, quality control, and statistical analyses is provided in the original papers. To create a PPI network of SCZ risk genes, we downloaded the STRING database [85] and extracted physically binding protein-protein interactions with a score ≥ 700, yielding a network of SCZ risk genes. We then only kept those nodes with *cis*-pGenes or *cis*-eGenes, yielding an SCZ network with 2011 nodes and 3118 protein-protein interactions. The generated network is used for calculating connectivity score.

### Prioritization analysis

We employed order statistics to integrate multiple datasets for prioritizing genes/proteins in GWAS loci [86, 87]. A total of 5 individual data sets with were used for this analysis: (1) pQTL data, ranked by the nominal *p* value; (2) eQTL data, ranked by the nominal *p* value; (3) colocalization between pQTLs and eQTLs; ranked by the PP_4_ values generated by the coloc program; (4) GeneCards disease-relevant score, ranked by the scores provided by GeneCards [88]; (5) Interaction with known SCZ-GWAS genes: ranked by the number of SCZ proteins and/or genes were connected to it. The final integrative protein ranking was generated by the order statistics.

## Supplementary information

Supplementary Information and Figures

Supplementary Tables

## Data Availability

The raw mass spectrometry data and RNA-seq from this study are available in the Synapse database under accession code syn32136022.1.

## Code availability

Data analyses were performed in LINUX shell, Perl (v5.18.4), and R (v4.0.4; https://www.r-project.org/). RNA-seq data were mapped to the human reference genome (GRCH37) using the following software tools: cutadapt (v1.12) (https://github.com/marcelm/cutadapt), STAR (2.4.2a) (https://github.com/alexdobin/STAR/releases), samtools (v1.3) (https://sourceforge.net/projects/samtools/files/samtools/1.3/), RSEM (v1.2.29) (https://github.com/deweylab/RSEM/releases/tag/v1.2.29). Genotype were processed by Eagle2 (https://alkesgroup.broadinstitute.org/Eagle/), Minimac3, (https://genome.sph.umich.edu/wiki/Minimac3). Annotation using ANNOVAR (https://annovar.openbioinformatics.org/en/latest/user-guide/startup/), Proteomic data were processed with JUMP software (https://github.com/JUMPSuite/JUMP). QTL mapping were performed using PEER (https://github.com/PMBio/peer), QTLtools (https://qtltools.github.io/qtltools/), coloc (https://github.com/cran/coloc), SMR (https://yanglab.westlake.edu.cn/software/smr/), and qvalue (https://github.com/StoreyLab/qvalue). STRING database for the network analysis (https://string-db.org/).
