# Supplementary material for: Genetic regulation of human brain proteome reveals proteins implicated in psychiatric disorders
Source: Mol Psychiatry. 2024 May 9;29(11):3330–43. doi: 10.1038/s41380-024-02576-8 (PMC11540848; doi:10.1038/s41380-024-02576-8)
Supplement: Supplementary file 1 — Supplementary Information and Figures [file 41380_2024_2576_MOESM1_ESM.docx]

**Supplementary Materials for**

**Genetic regulation of human brain proteome uncovers proteins implicated in psychiatric disorders**

Jie Luo^1, *^, Ling Li^2, *^, Mingming Niu^3, *^, Dehui Kong^2^, Yi Jiang^4^, Suresh Poudel^5^, Annie W. Shieh^6^, Lijun Cheng^6^, Gina Giase^6^, Kay Grennan^6^, Kevin P. White^7^, Chao Chen^8^, Sidney H. Wang^9^, Dalila Pinto^10^, Yue Wang^11^, Chunyu Liu^12, †^, Junmin Peng^3, †^, Xusheng Wang^2,5 †^

1. State Key Laboratory for Managing Biotic and Chemical Threats to the Quality and Safety of Agro‐products, Zhejiang Academy of Agricultural Sciences, Hangzhou, Zhejiang 310021, China
2. Department of Genetics, Genomics & Informatics, University of Tennessee Health Science Center, Memphis, TN 38103, USA
3. Department of Structural Biology, St. Jude Children’s Research Hospital, Memphis, TN 38105, USA
4. Department of Epidemiology and Biostatistics, School of Public Health, Tongji Medical College, Huazhong University of Science and Technology, Wuhan, Hubei 430030, China
5. Center for Proteomics and Metabolomics, St. Jude Children's Research Hospital, Memphis, TN 38105, USA
6. Knapp Center for Biomedical Discovery, University of Chicago, Chicago, IL 60637, USA
7. Department of Biochemistry and Precision Medicine, National University Singapore, 119077, Singapore
8. Center for Medical Genetics and Human Key Laboratory of Medical Genetics, School of Life Sciences, Central South University, Changsha, Hunan 410083, China
9. Center for Human Genetics, Brown Foundation Institute of Molecular Medicine, The University of Texas Health Science Center at Houston, Houston, TX 77225, USA
10. Department of Genetics and Genomic Sciences, Icahn School of Medicine at Mount Sinai, New York, NY 10029, USA
11. Department of Electrical and Computer Engineering, Virginia Polytechnic Institute and State University, Arlington, VA 22203, USA
12. Department of Psychiatry, SUNY Upstate Medical University, Syracuse, NY 13210, USA

**Supplementary Information**

**Impacts of confounding factors on the detection of pQTLs and eQTLs**

To assess the impact of known factors on the detection of pQTLs and eQTLs, we performed four evaluations. Firstly, we conducted a correlation analysis between PEER factors and known technical and biological covariates in both transcriptomics and proteomics data. We included six common covariates in both datasets, namely disease groups, brain bank, gender, age of death, post-mortem interval (PMI), and ethnicity. Additionally, we included proteomic data-specific covariate, which was the quality of protein samples (i.e., whether they contained blood contaminant), and transcriptomic data-specific covariates, which comprised RNA integrity number (RIN), and RNA library. The analysis indicated that PEER factors from transcriptomics and proteomics data were significantly correlated with several known technical and biological covariates (**Supplementary Figure 2A** and **Supplementary Figure 4A**), indicating that the technical and biological covariates were removed by the PEER analysis. In the proteomics data, we identified significant correlations between PEER factors and various factors, including sample quality, disease group, brain bank, age of death, and PMI. However, we observed weaker correlations between PEER factors and gender and ethnicity. Similarly, in the transcriptomics data, we observed strong correlations between PEER factors and brain bank, age of death, and RIN, but weaker correlations with disease groups, sex, PMI, and RNA library. Secondly, the correlation analysis between known factors and gene/protein expression also suggests that the effects of known co-variates were removed from both transcriptomics and proteomics data (**Supplementary Figure 2B** and **Supplementary Figure 4B**). To evaluate the PEER correction, we used the distributions of the correlation *p*-values between a specific covariate and all proteins/transcripts before and after the correction. The Kolmogorov-Smirnov (KS) test was used for statistical testing. The results showed that for proteomics data, several known factors including disease groups, brain bank, age of death, and PMI were highly correlated before the PEER analysis, but p-values are evenly distributed after the PEER analysis (**Supplementary Figure 2B**). Similar results are obtained for transcriptomics data (**Supplementary Figure 4B**). In summary, the *p* value distributions obtained from modeling the covariates strongly suggest that adjusting for them would improve the downstream analysis steps. Thirdly, QTL mapping with PEER corrections shows that the PEER correction increases the number of pQTLs and eQTLs compared to without PEER corrections (**Supplementary Figure 2C** and **Supplementary Figure 4C**). Furthermore, including known co-variates in addition to the PEER factors did not detect additional pQTLs and eQTLs (**Supplementary Figure 2D** and **Supplementary Figure 4D**). All the above four analyses indicate that PEER corrections for both proteomics and transcriptomics data had removed the effects of known and hidden co-variates.

**Performance Comparison Between JUMP and MSFragger**

Mass spectrometry raw data were analyzed using the JUMP search engine, which is designed to improve the sensitivity and specificity of results. Numerous software tools, including Proteome Discoverer, MaxQuant, and MSFragger, have been developed for peptide identification. To validate the confidence in peptide identification by JUMP, we performed a comprehensive comparison between JUMP and MSFragger. This comparison was conducted using a batch of 11-plex TMT data generated in this study. The data were analyzed by both software tools, and identifications were filtered at a protein FDR of less than 1%. A vast majority (97.1%; 8,421/8,670) of proteins detected by JUMP were also identified by MSFragger (**Supplementary Figure 16A**). Similarly, 96.0% of peptides and 92.1% of PSMs identified by JUMP were corroborated by MSFragger (**Supplementary Figure 16B,C**). Although MSFragger identified a higher number of proteins, those exclusively identified by MSFragger tended to be less confident compared to those identified by JUMP. Among these exclusively identified proteins by JUMP and MSFragger, 33.3% of the proteins identified by JUMP were supported by only one PSM, whereas 52.7% of the proteins identified by MSFragger were supported by only one PSM (**Supplementary Figure 16D**). To further examine the quality of these identifications by JUMP and MSFragger, we analyzed the scoring distributions of JUMP's JScore and MSFragger's Hyperscore, particularly focusing on those identifications with a single PSM. Our findings reveal that proteins with a single PSM identified by JUMP maintained a JScore distribution comparable to that of proteins identified by multiple PSMs. In contrast, MSFragger's single PSM identifications typically exhibited lower scores relative to its multiple PSMs (**Supplementary Figure 16E, F**). After excluding peptides and proteins with more than one PSM, 98% of proteins (8,209 out of 8,375) detected by JUMP were also identified by MSFragger (**Supplementary Figure 16G**). Similarly, 93% of peptides identified by JUMP were corroborated by MSFragger (**Supplementary Figure 16H**). This overlap in peptide identification could increase with the inclusion of additional data batches.

**Supplementary Figures**

**Supplementary Figure 1**. Functional terms enriched in highly and lowly variable proteins.

**Supplementary Figure 2**. Impacts of confounding factors in pQTL analysis.

**Supplementary Figure 3**. Workflow of the linkage analysis of genome-wide transcriptome and proteome.

**Supplementary Figure 4**. Impacts of confounding factors in eQTL analysis.

**Supplementary Figure 5**. Summary of QTL mapping of gene expression.

**Supplementary Figure 6**. Influence of Protein-Altering Variants (PAVs) on the regulation of protein expression.

**Supplementary Figure 7**. Circos plot illustrating 10 *trans*-QTLs with more than 5 regulated proteins.

**Supplementary Figure 8**. Analysis of the colocalization of *cis*-pQTLs, *cis*-eQTLs, and GWAS loci.

**Supplementary Figure 9**. Mediation analysis of colocalized *cis*-pQTLs and *cis*-eQTLs.

**Supplementary Figure 10**. Forest plots showing the effect size of 4 GWAS loci causally controlled by eGenes.

**Supplementary Figure 11**. Network analysis for SCZ GWAS risk genes.

**Supplementary Figure 12**. Heatmap showing cell-type-specific differential expression and abundance of the top 60 ranked proteins.

**Supplementary Figure 13**. Comparison of the effect sizes between *cis*-eQTLs and *cis*-pQTLs.

**Supplementary Figure 14**. Comparison of *cis*-pQTLs detected by QTLtools without population structure and by GEMMA with population structure.

**Supplementary Figure 15.** Impact of low expression on *cis*-eQTLs detection.

**Supplementary Figure 16.** Comparison of peptide identifications by JUMP and MSFragger.

**Supplementary Tables**

**Supplementary Table 1A**. Demographic information and clinical characteristics of human subjects used in the proteomics experiment.

**Supplementary Table 1B**. Summary of clinical characteristics of human subjects and information of the proteomics experiment.

**Supplementary Table 1C**. Demographic information and clinical characteristics of human subjects used in the transcriptomics experiment.

**Supplementary Table 1D**. Summary of clinical characteristics of human subjects and information of transcriptomics profiling.

**Supplementary Table 2**. Proteome profiling of human brain cortex tissues by TMT-LC/LC-MS/MS.

**Supplementary Table 3**. Transcriptome profiling of human brain cortex tissues by RNA-seq.

**Supplementary Table 4A**. Significant *cis*-pQTLs detected by proteome-wide association analysis in the brain cortex tissue from 268 individuals.

**Supplementary Table 4B**. Significant *trans*-pQTLs identified by proteome-wide association analysis in the brain cortex tissue from 268 individuals.

**Supplementary Table 5A**. Significant *cis*-eQTL identified by transcriptome-wide association analysis in the brain cortex tissue from 416 individuals.

**Supplementary Table 5B**. Significant *trans*-eQTLs identified by transcriptome-wide association analysis in the brain cortex tissue from 416 individuals.

**Supplementary Table 6.** Influence of Protein-Altering Variants (PAVs) on the regulation of protein expression.

**Supplementary Table 7A**. Co-localization analysis for *cis*-pQTL and *cis*-eQTL.

**Supplementary Table 7B**. Co-localization analysis for *cis*-pQTL and SCZ GWAS.

**Supplementary Table 7C**. Co-localization analysis for *cis*-pQTL and BP GWAS.

**Supplementary Table 7D**. Co-localization analysis for *cis*-eQTL and SCZ GWAS.

**Supplementary Table 7E**. Co-localization analysis for *cis*-eQTL and BP GWAS.

**Supplementary Table 8**. Transcript-independent and transcript-dependent regulations identified by mediation analysis.

**Supplementary Table 9A**. SMR analysis result with summary statistics of SCZ GWAS meta-analysis using protein expression as the exposure.

**Supplementary Table 9B**. SMR analysis result with summary statistics of SCZ GWAS meta-analysis using gene expression as the exposure.

**Supplementary Table 9C**. SMR analysis result with summary statistics of BP GWAS meta-analysis using gene expression as the exposure.

**Supplementary Table 10**. Integrating multi-omic data sets to prioritize candidate proteins for SCZ GWAS loci.

**Supplementary Table 11.** Spectral validation for low-confidence peptide-spectrum-matches (PSMs).

**Supplementary Table 12**. SCZ risk genes collected from 9 published papers.

**
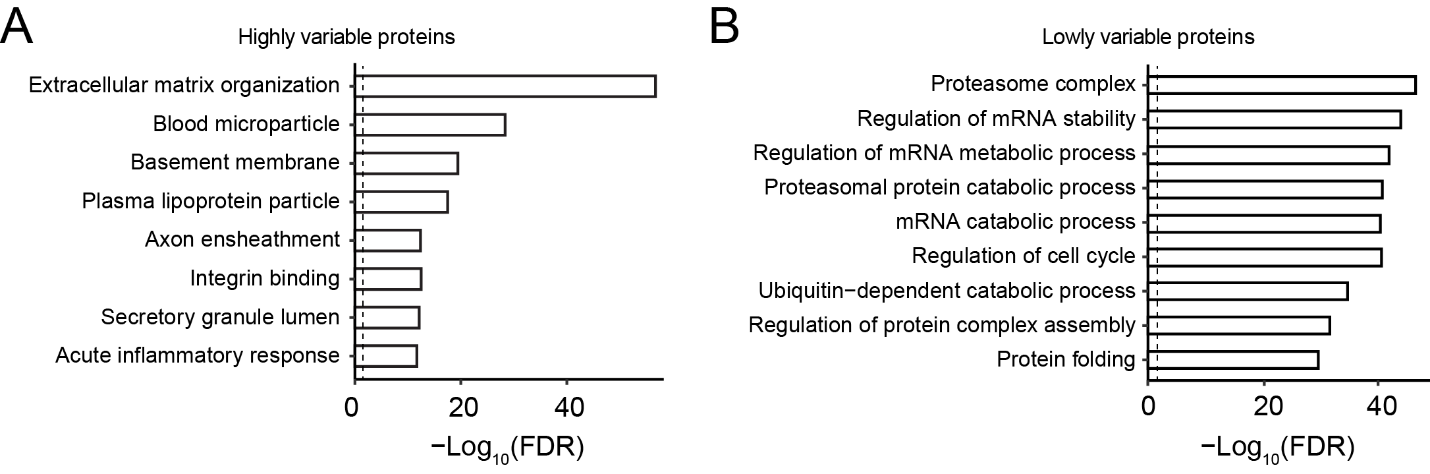
**

**Supplementary Figure 1. Functional terms enriched in highly and lowly variable proteins.**

**
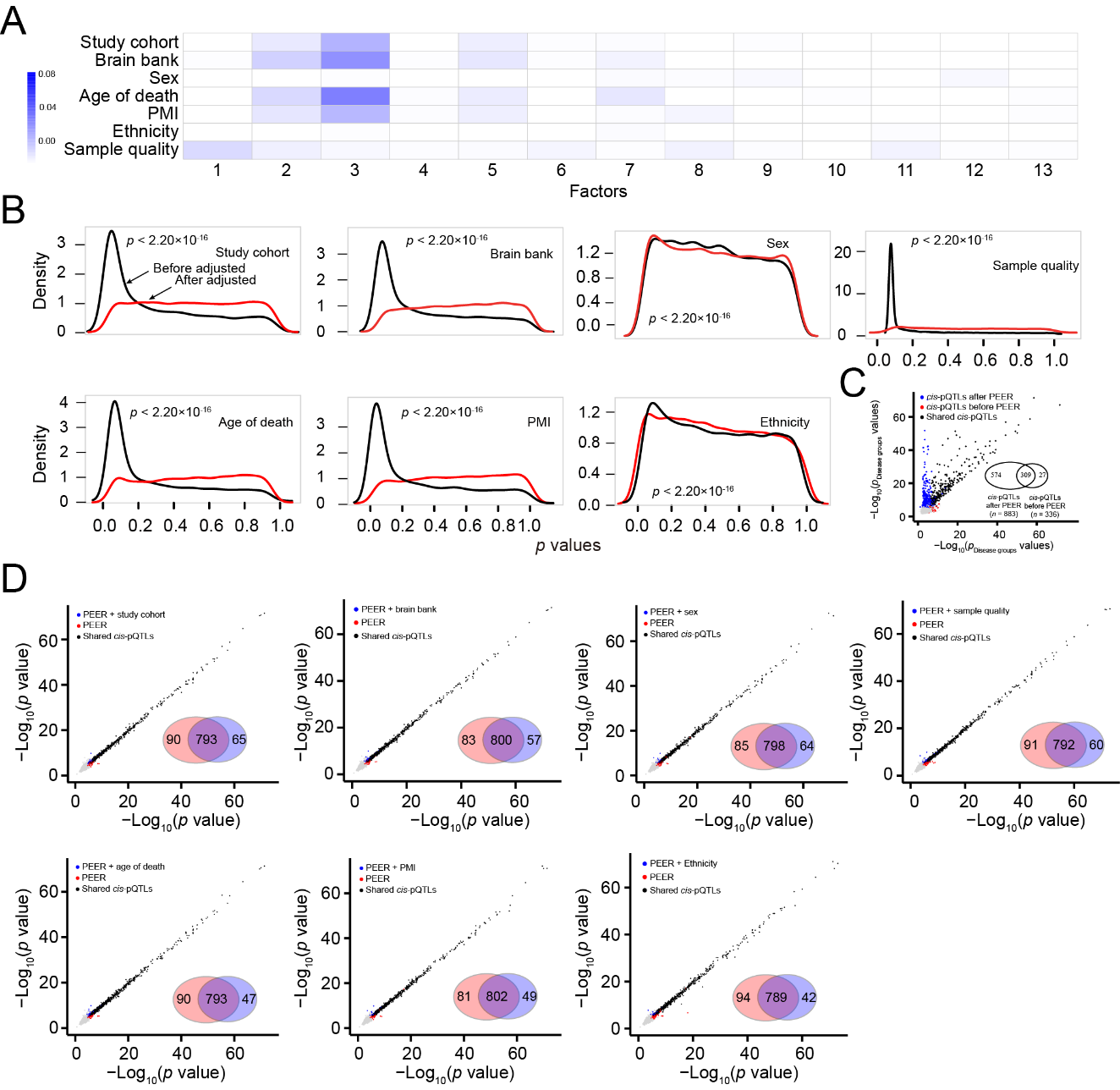
**

**Supplementary Figure 2. Impacts of confounding factors in pQTL analysis.** **A** Heatmap showing the correlation between seven known confounding variables and PEER factors in proteomics data. The seven known confounding factors include study cohort, brain bank; sex; age of death, post-mortem interval (PMI), ethnicity, and sample quality. The sample quality is a metric indicating the presence of blood contamination in a sample. **B** Density plots displaying the distribution of correlation between seven confounding factors and protein expression levels before (black) and after (red) the PEER correction. **C** Scatter plots showing the distribution of *cis*-pQTL *p* values before and after PEER analysis. **D** Scatter plots showing *cis*-pQTL *p* values without/with a confounding co-variate in addition to PEER factors.

**
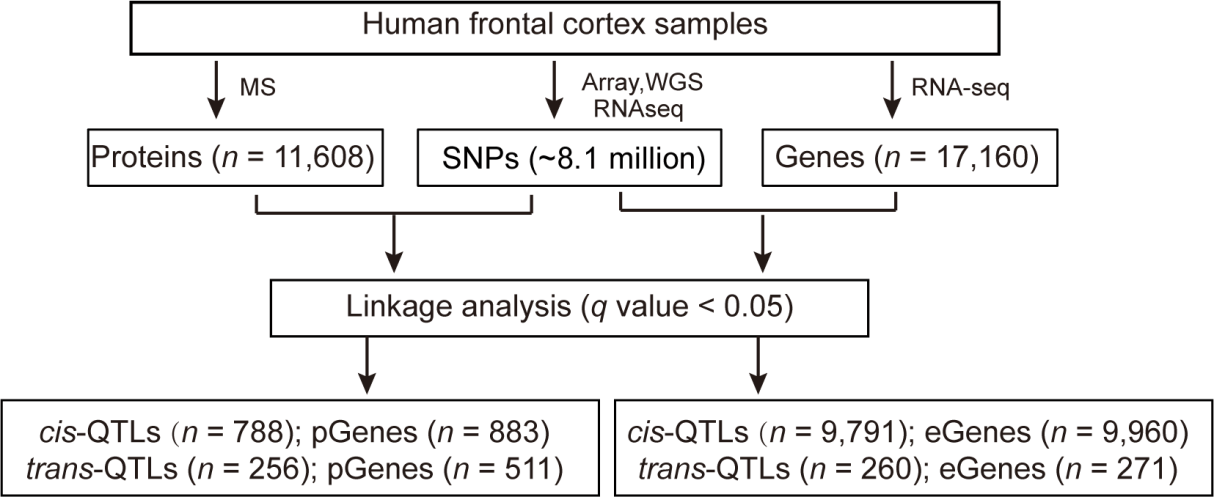
**

**Supplementary Figure 3. Workflow of the linkage analysis of genome-wide transcriptome and proteome.**

**
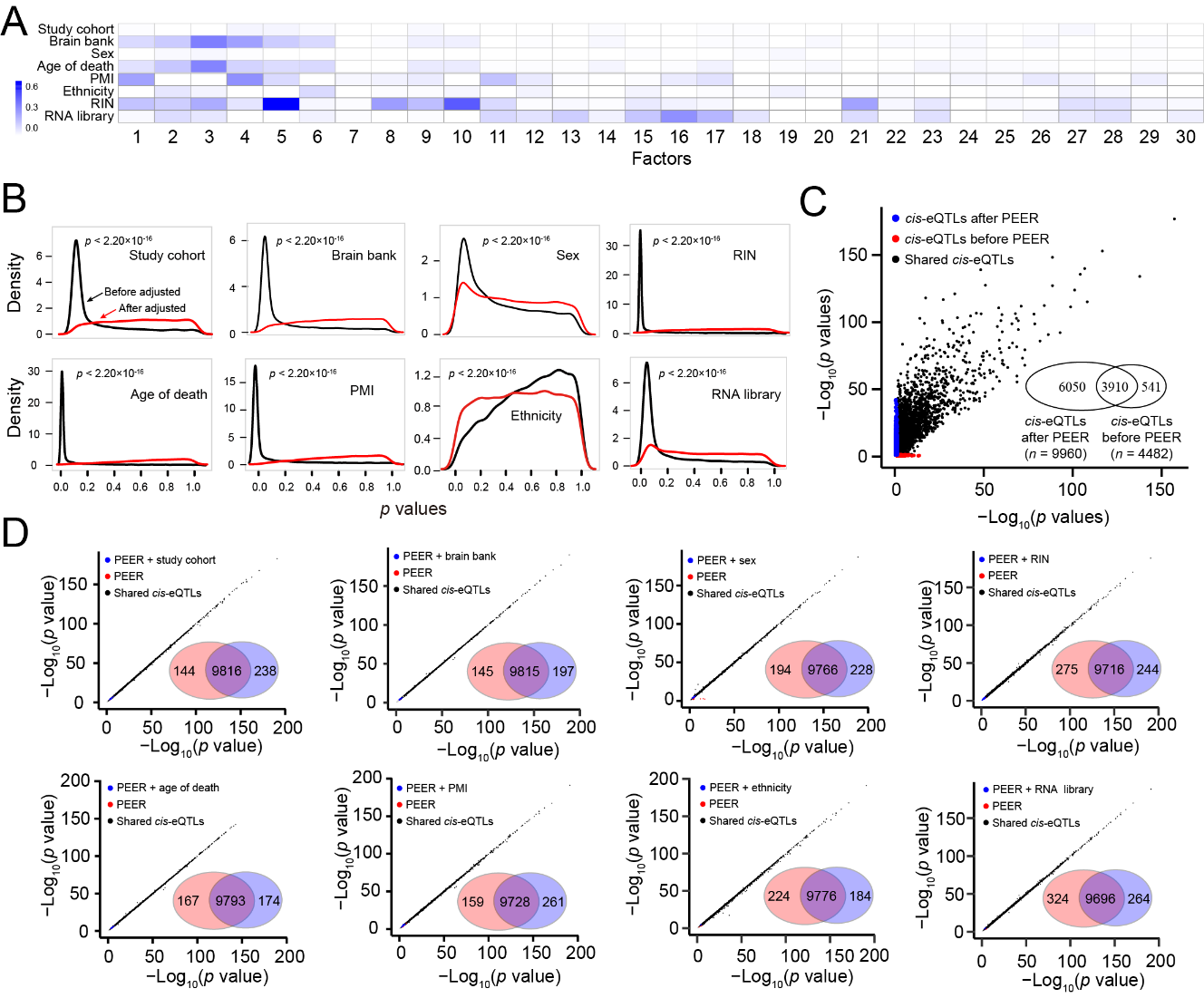
**

**Supplementary Figure 4. Impacts of confounding factors in eQTL analysis.** **A** Heatmap showing the correlation between seven known confounding variables and PEER factors in proteomics data. The eight known confounding factors include study cohort; brain bank; sex; RIN, RNA library, age of death, post-mortem interval (PMI) and ethnicity. The sample quality is a metric indicating the presence of blood contamination in a sample. **B** Density plots displaying the distribution of correlation between seven confounding factors and protein expression levels before (black) and after (red) the PEER correction. **C** Scatter plots showing the distribution of *cis*-eQTL *p* values before and after PEER analysis. **D** Scatter plots showing *cis*-eQTL *p* values without/with a confounding co-variate in addition to PEER factors.


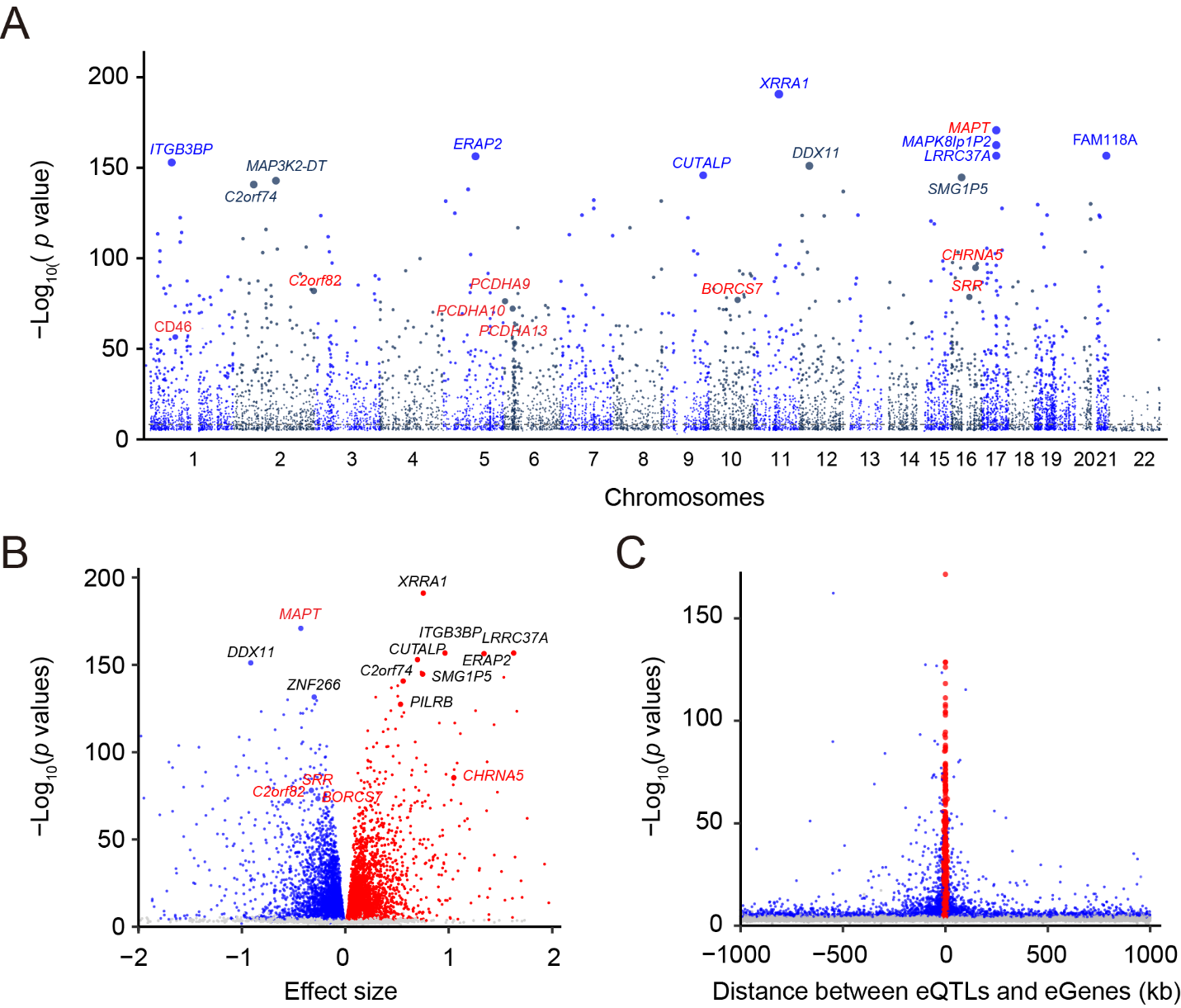


**Supplementary Figure 5. Summary of QTL mapping of gene expression. A** Manhattan plot for proteome-wide association analysis. The -log_10_ *p* values for association with protein expression of SNPs are plotted by genomic position with chromosome number listed across the bottom. The *y*-axis shows -log_10_ *p* value for association with protein expression. **B** Relationship between effect size and *p* values for 9,791 *cis*-eQTLs. The *x*-axis is the effect size and the *y*-axis is -Log_10_ *p* value. **C** The relationship between the distance to *cis*-eQTLs and significance level (-log_10_ *p* value). The distance to eQTLs was defined as the distance to the transcriptional start site of the genes showing eQTLs.


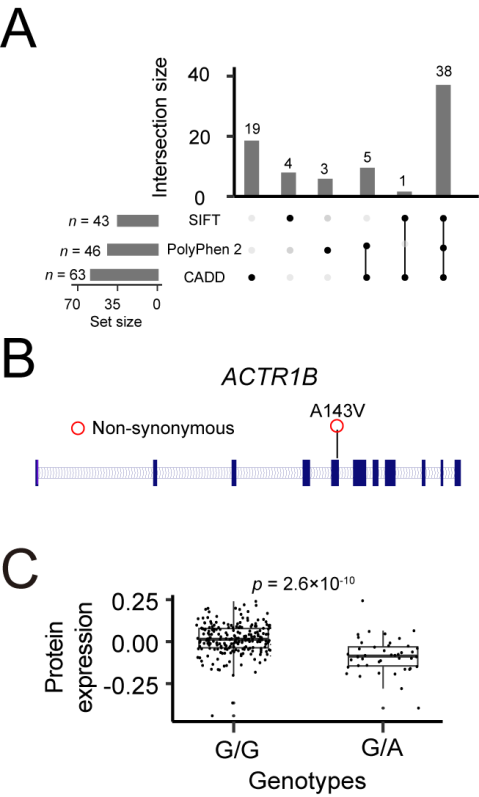


**Supplementary Figure 6. Influence of Protein-Altering Variants (PAVs) on the regulation of protein expression. A** Venn diagram displaying the number of non-synonymous variants predicted as deleterious by three methods: CADD, SIFT, and PolyPhen 2. **B** An example of a deleterious mutation (A143V) found in the ACTR1B protein. **C** Box plot showing normalized ACTR1B protein expression and *rs1162435* allele dosage.

**
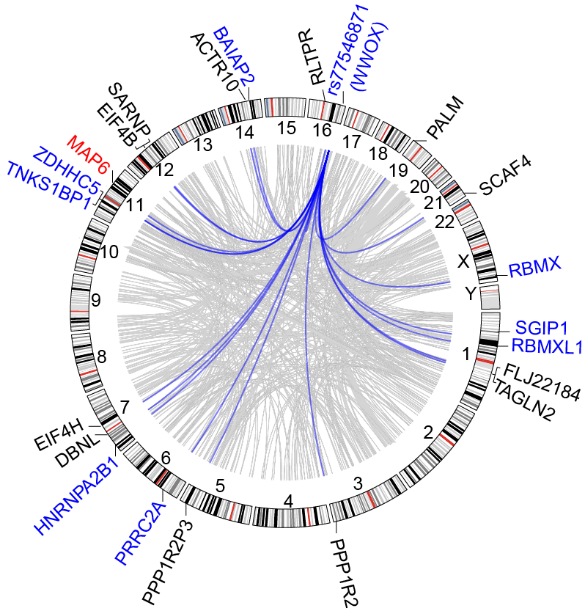
**

**Supplementary Figure 7. Circos plot illustrating 10 trans-QTLs with more than 5 regulated proteins.** The inner blue lines of the circos plot represent a *trans*-pQTL, *rs77546871*, mapped to WWOX protein.

**
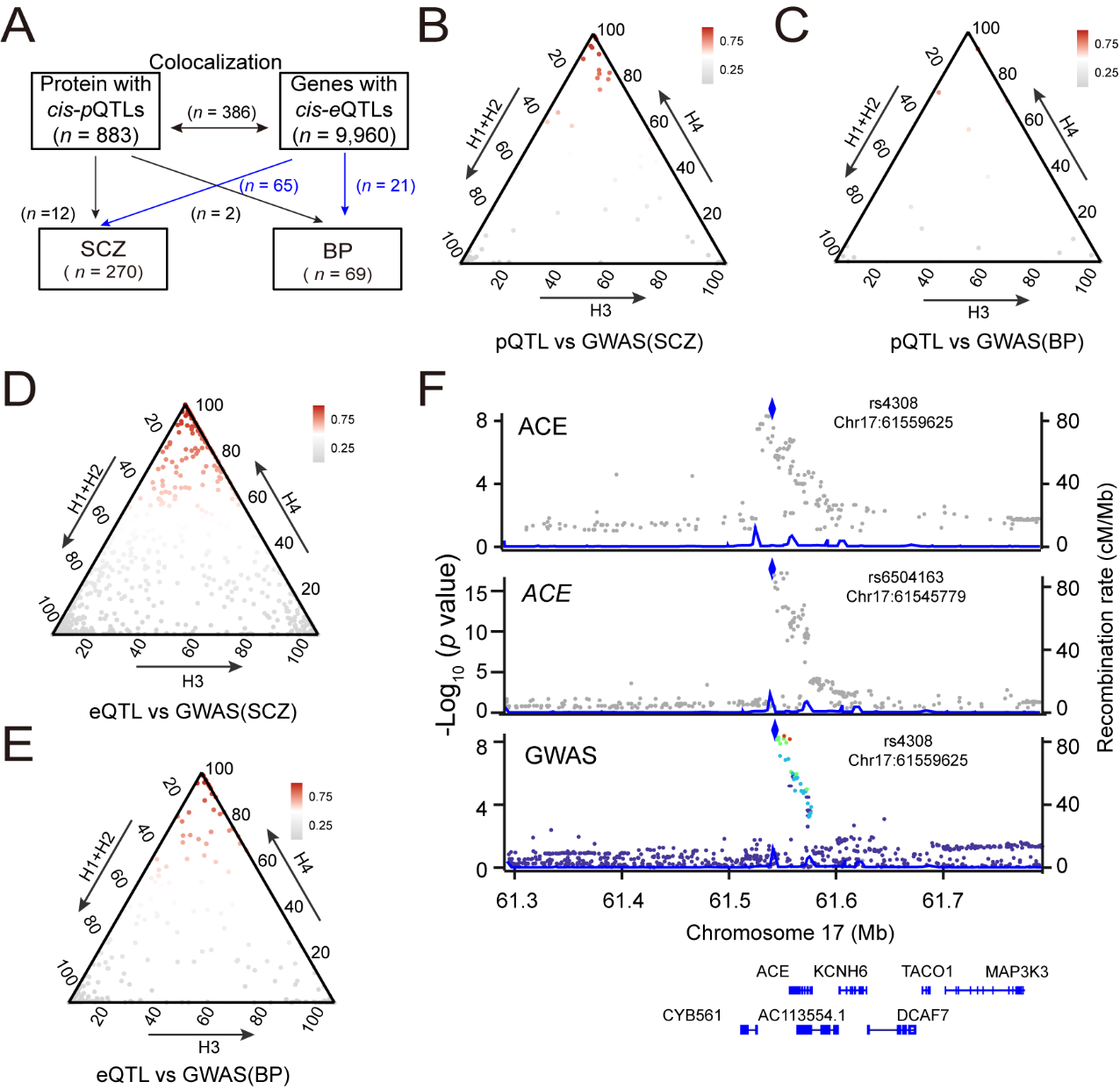
**

**Supplementary Figure 8. Analysis of the colocalization of *cis*-pQTLs, *cis*-eQTLs, and GWAS loci.** **A** Diagram showing the numbers of colocalized *cis*-pQTLs, *cis*-eQTLs, and GWAS loci. **B, C** Ternary plot showing colocalization posterior probabilities of cis-pQTLs, SCZ GWAS loci (B), and BP GWAS loci (C). **D, E** Ternary plot showing colocalization posterior probabilities of *cis*-eQTLs, SCZ GWAS loci (D), and BP GWAS loci (E). **F** LocusZoom plot showing the colocalization of *cis*-pQTL, *cis*-eQTL, and SCZ GWAS loci.

**
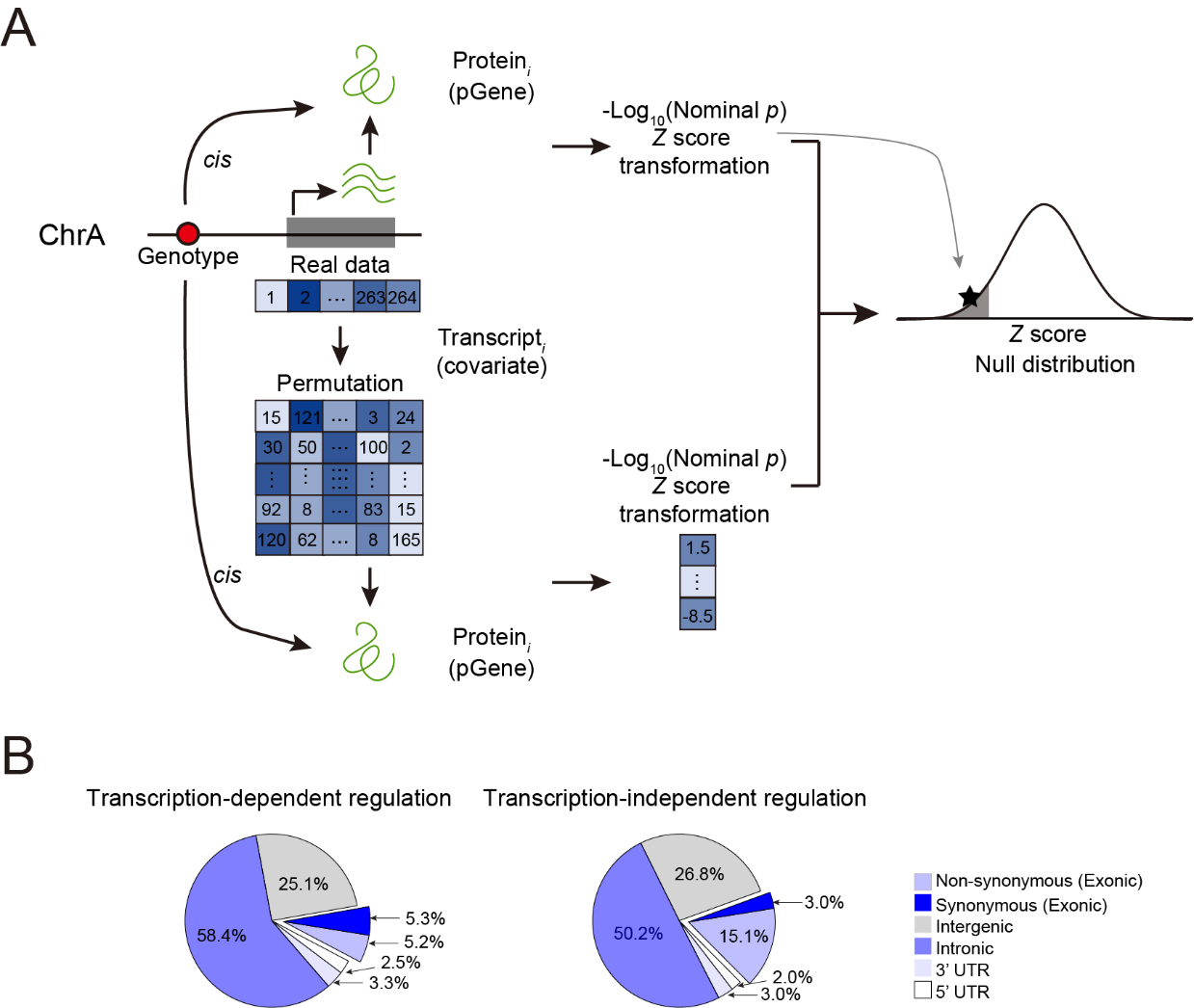
**

**Supplementary Figure 9. Mediation analysis of colocalized *cis*-pQTLs and *cis*-eQTLs. A** Schematic diagram showing the mediation analysis. We use conditional mapping to identify eGenes that are likely to be a causal mediator between the pQTL and protein expression it regulates. To assess whether the *p*-value significantly drops for a given mediator on a *cis*-pQTL, a null distribution of *p* values is estimated by randomly permuting sample labeling of the eGene. A total of 1,000 permutations was used to generate null distribution. The mapping *p*-values are then converted into z-scores. We consider a potential mediator with a z-score ≤ -4.26. **B** Pie chart showing the distribution of *cis*-pQTLs in different genomic regions between transcription-dependent and transcription-independent regulation.

**
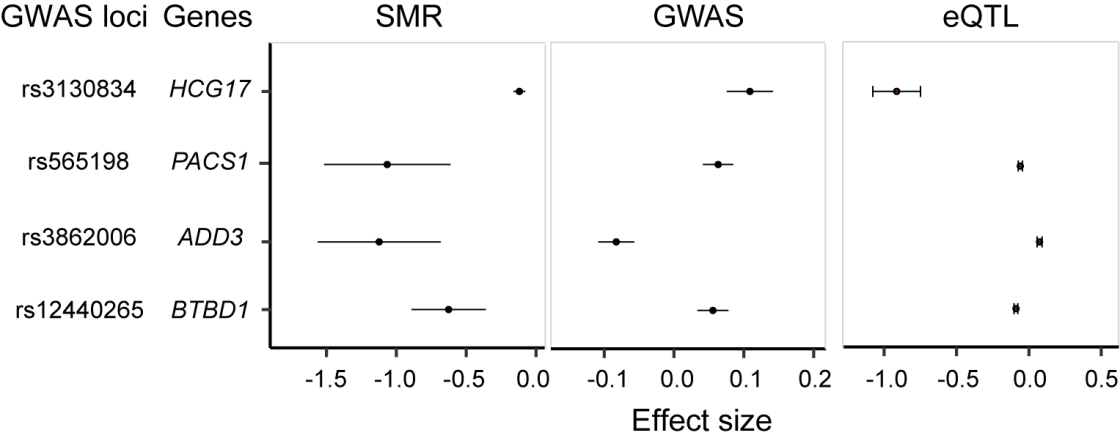
**

**Supplementary Figure 10. Forest plots showing effect sizes of 4 BP GWAS loci causally controlled by eGenes, respectively.** The causality relationship was estimated by the SMR/HEIDI method. Center values mark effect size point estimates, and error bars represnet the 95% confidence intervals.

**
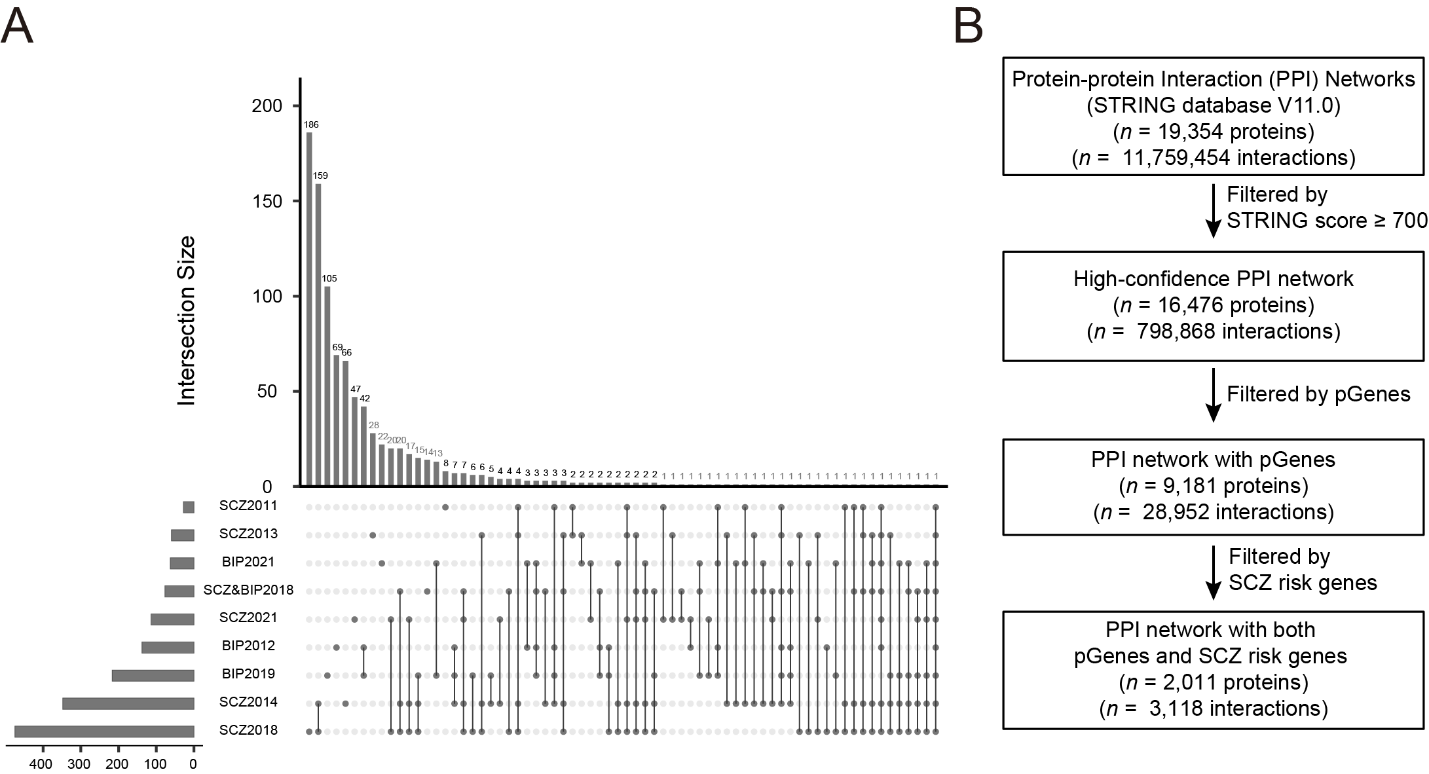
**

**Supplementary Figure 11. Network analysis for SCZ GWAS risk genes. A** UpSet plot showing intersections of SCZ risk genes collected from different GWA studies. **B** Flow chart showing the network analysis. Protein-protein interaction network was downloaded from the STRING database (https://string-db.org; version 11.0) and highly confident protein-protein interactions with a score ≥ 700 were extracted. We then extracted interactions with nodes that are either pGenes or SCZ GWAS risk genes, resulting in a PPI network with 2,011 nodes and 3,118 edges.

**
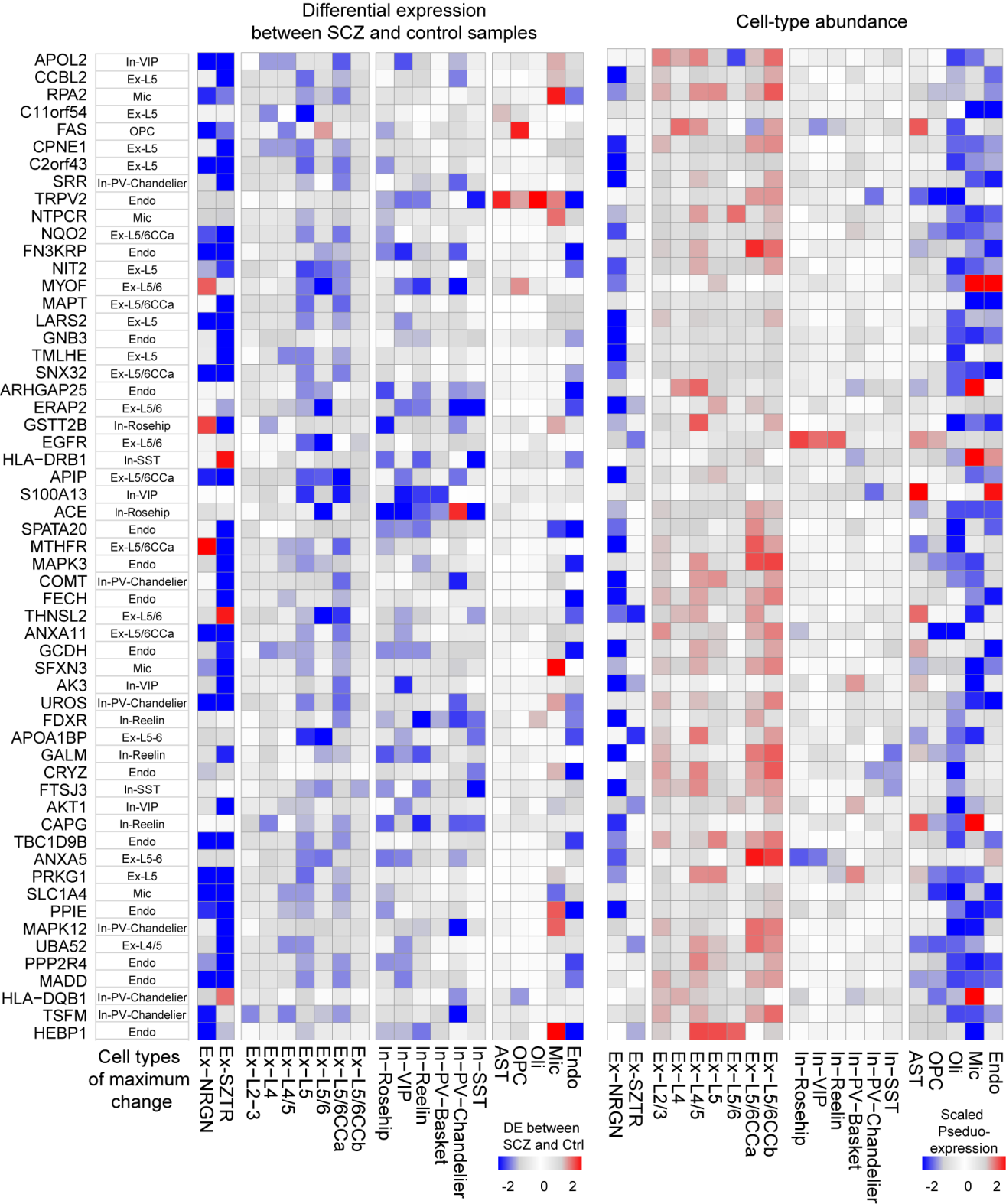
**

**Supplementary Figure 12. Heatmap showing cell-type-specific differential expression and abundance of the top 60 ranked proteins**. The cell-type-specific expression data were downloaded from a recent single-cell transcriptomic data. The cell types of maximum change between SCZ and control samples are shown on the left. A heatmap showing log_2_(fold change) between SCZ and Ctrl for each cell type (middle); A heatmap showing expression abundance in each cell type (right). The expression abundance in each cell type was summed across all cells and samples.

**
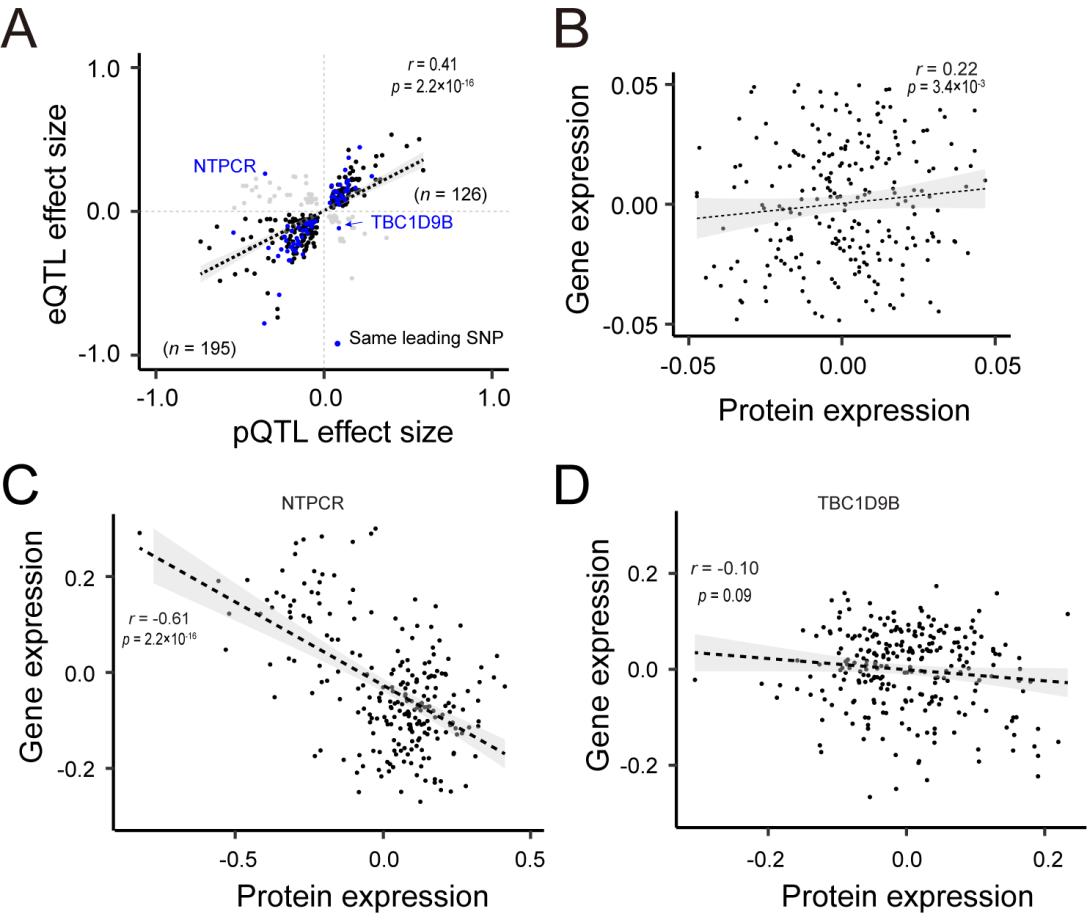
**

**Supplementary Figure 13. Comparison of the effect sizes between *cis*-eQTLs and *cis*-pQTLs.** **A** Scatter plot showing the distribution of effect sizes of colocalized *cis*-eQTLs and *cis*-pQTLs. **B** Correlation of expression levels of colocalized *cis*-eGenes and *cis*-pGenes. **C-D** Scatter plot showing three pGenes that had an inconsistent direction of *cis*-pQTL and *cis*-eQTL. These two genes/proteins were mapped as *cis*-eQTL and *cis*-pQTL, but the inconsistent direction of effect.

**
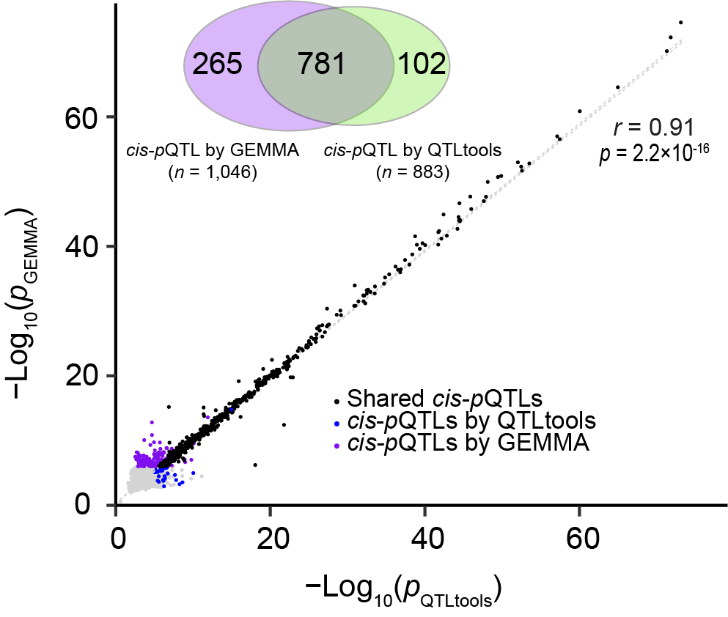
**

**Supplementary Figure 14. Comparison of *cis*-pQTLs detected by QTLtools without population structure and by GEMMA with population structure.** The Venn diagram shows 88% (781/883) of significant cis-pGenes identified by both QTLtools and GEMMA analyses.

**
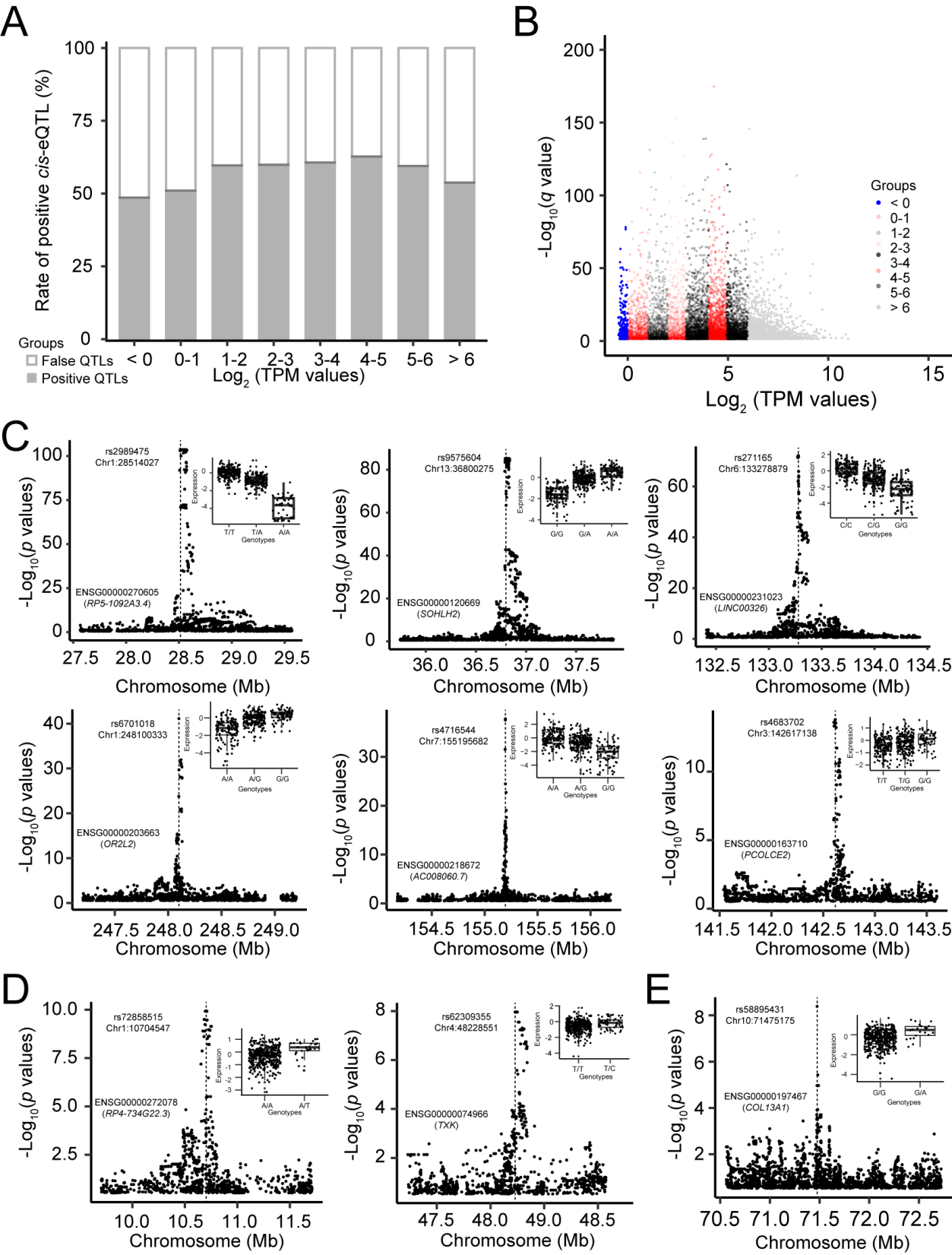
**

**Supplementary Figure 15. Impact of low expression on *cis*-eQTLs detection.** **A** Stacked bar plot showing the rate of *cis*-eQTLs across different expression levels. **B** Manhattan plots displaying *cis*-eQTLs whose genes exhibited low expression levels (TPM < 1; Log_2_ (TPM) < 0). **C** Manhattan plots displaying six examples with higher q values but lower expression values for *cis*-eQTLs. **D** Manhattan plots showing two examples of *cis*-eQTLs with two genotypes. **E** Manhattan plot displaying an example of a *cis*-eQTL with two genotypes but low confidence.


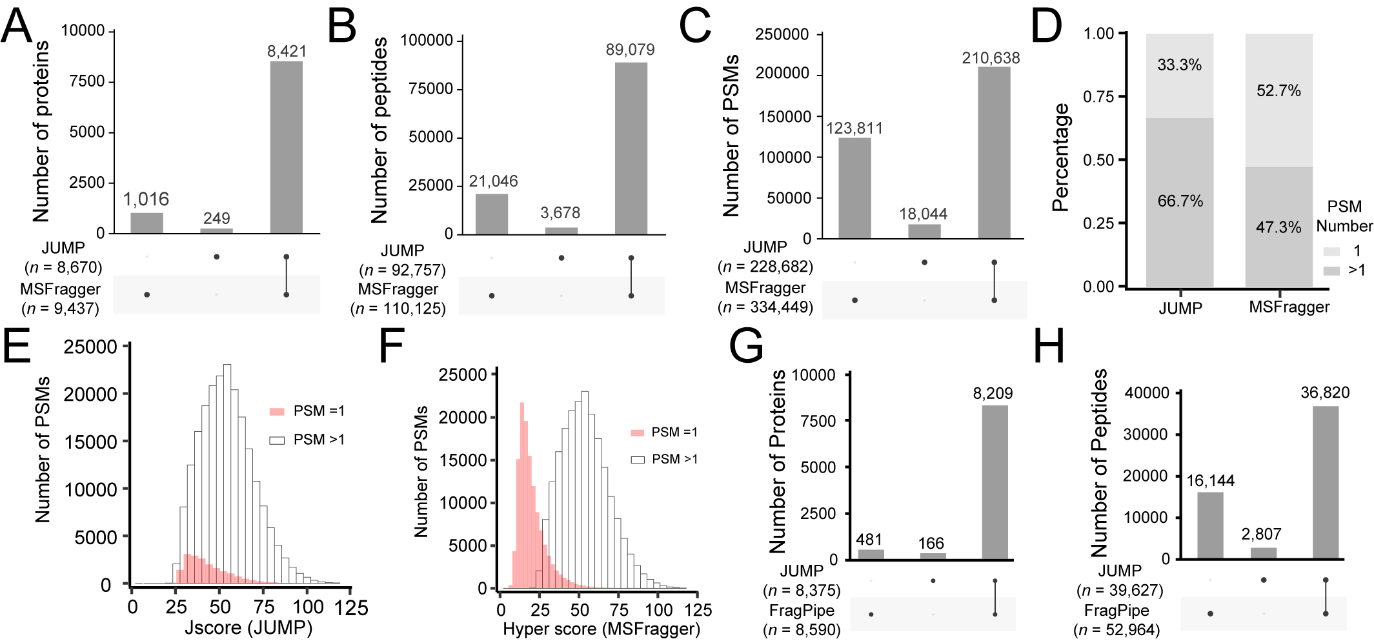


**Supplementary Figure 16. Comparison of Identifications Between JUMP and MSFragger.** (A-C) UpSet plots illustrate the identification of proteins (A), peptides (B), and peptide-spectrum matches (PSMs) (C) by JUMP and MSFragger, highlighting the number of identifications exclusive to each tool and those shared between them. (D) Stacked bar charts show the proportion of proteins with either a single PSM or multiple PSMs, identified exclusively by either JUMP or MSFragger. (E) Distribution of Jscore from JUMP analysis, with PSMs grouped by whether the Jscore is equal to 1 or greater than 1. The bars highlighted in light red represent identifications of 1 PSM. (F) Distribution of Hyper scores from MSFragger analysis, categorized by PSMs with a Hyper score of 1 and those with scores greater than 1. The bars highlighted in light red represent identifications of 1 PSM. (G-H) UpSet plots display the identification of proteins (G) and peptides (H) for those proteins and peptides associated with more than 1 PSM.
